# Control over Recommendation Algorithms in Heterogeneous Modular Systems with Dynamic Opinions

**DOI:** 10.3390/e28030333

**Published:** 2026-03-16

**Authors:** Vladislav Gezha, Ivan Kozitsin

**Affiliations:** Laboratory of Active Systems, V. A. Trapeznikov Institute of Control Sciences of Russian Academy of Sciences, 117997 Moscow, Russia; gezha.vn@phystech.edu

**Keywords:** opinion dynamics, mean-field approximation, ranking algorithms, modular networks, optimal control, finite-difference schemes

## Abstract

The paper suggests a model-dependent theoretical framework for designing optimal ranking algorithms to achieve desirable macroscopic opinion configurations. We consider an opinion formation process in which agents communicate through stochastic pairwise interactions, with the outcomes of these interactions being a function of the interacting agents’ opinions and individual attributes (types). For the model, we write a mean-field approximation (MFA)—a coarse-grained nonlinear ordinary differential equation—which accommodates network modularity and assortativity, agents’ activity heterogeneity, and the curation of a ranking system that can prohibit interactions with opinion- and type-dependent probabilities. Upon MFA, we formulate a control problem for dynamically adjusting the ranking algorithm’s parameters. The existence of a solution is proved, and certain properties of optimal controllers are derived. For the case of a two-element opinion alphabet, we obtain a solution to the control problem using finite-difference schemes. This solution holds for any number of agent types and does not depend on external factors, such as the influence of social bots. Numerical tests corroborate our findings and also enable us to investigate the control problem for high-dimension opinion spaces, wherein we consider two primary scenarios: depolarization of an initially polarized society and nudging a social system towards a fixed endpoint of an opinion spectrum.

## 1. Introduction

In the era of digitalization and information overload, it is extremely important to understand not only how individuals process new information but also how they acquire it. Despite the first question being the subject of analysis by scholars since the mid-20th century—beginning with the seminal work of French [1]—the second question has received relatively little attention in the literature. The problem is that, in the digital domain, individuals acquire new information under the curation of ranking algorithms (aka recommendation, personalization, or filtering systems) [2]. These algorithms leverage users’ data and provide them with content that aligns with their preferences, amplifies engagement, and perhaps subtly affects their opinions [3].

The mainstream of research on recommendation systems concerns how these technologies affect opinion polarization and the formation of echo-chambers on social platforms [4]. In general, the literature suggests that ranking algorithms contribute to polarization and information bubbles by forming feedback loops in which users are repeatedly exposed to content that confirms their views, thereby inducing positive reactions that are subsequently acknowledged by the system, and so on [5].

However, ranking systems can be applied to achieve completely opposite effects, such as depolarization [6]. In this article, we formulate, investigate, and solve, by means of analytical derivations and numerical experiments, a control problem in which one dynamically adjusts a ranking algorithm to sway the opinions of individuals according to a predefined objective. For instance, this objective can embrace the strive for depolarization. Our control problem is model-dependent. We build upon an opinion formation model [7,8], which effectively combines information on agents’ types, their activity heterogeneity, and the mesoscopic and macroscopic properties of the underlying networks. The model is able to span different mechanisms of social influence, such as assimilation or bounded confidence [9], thus providing a flexible framework for studying control over ranking algorithms.

## 2. Backgrounds

Our study lies at the intersection of two closely related areas of research: (i) opinion formation modeling and (ii) recommendation system modeling. To motivate the subject of our study, we begin by briefly reviewing opinion formation models.

### 2.1. Opinion Formation Models

Opinion formation (aka opinion dynamics) models consider a population of agents, possibly immersed in a social graph. These agents interact with each other according to a predefined set of rules and update their opinions following these microscopic interactions [10,11]. Agents’ opinions are typically described by numerical quantities, either discrete [12,13] or continuous [14]. In a typical case, these interactions lead to opinion assimilation, with more “distant” opinions ensuring greater assimilation [10]. However, psychological studies indicate that individuals with outlying opinions rarely listen to each other’s arguments, a phenomenon that is captured in bounded confidence models [15,16,17]. Finally, in some cases, communications between contrary opinions may give rise to opinion dissimilation, when opinions further diverge [9,18].

Empirical studies of social influence and opinion formation in social groups report rather mixed evidence. Laboratory experiments typically confirm the mechanism of opinion assimilation [19], whereas observational studies in online networks lend support for bounded confidence and dissimilation [20,21,22].

We refer the interested Reader to the excellent review articles in [16,17,23,24,25] for more information on the current state of the art in the field of modeling social influence and opinion formation in social networks.

What we would like to highlight here is that it was the opinion formation models underlying the analysis of ranking algorithms and their influence on society in the earliest studies [4,26]. The reason is that these models provide a flexible framework that can be easily adapted to incorporate the mechanics of recommendation systems into the models’ protocols.

### 2.2. Ranking Algorithms: A General View

Ranking algorithms are programs that curate the information users observe in their news feeds on social media platforms. Considering individuals’ limited cognitive and time resources [27], these algorithms help navigate users in the online domain by selecting the most relevant content. However, there are serious concerns that ranking algorithms, while addressing their own—essentially hidden—objectives, contribute to opinion polarization and the formation of echo chambers [2].

In fact, there are various forms of such algorithms that have slightly different purposes: some filter content in news feeds (filtering algorithms), while others recommend new acquaintances (recommendation algorithms). In turn, personalization typically refers to providing a user with content that aligns with that user’s individual preferences. To avoid any confusion, in what follows, we will use all these terms interchangeably whenever possible.

The four main principles of filtering are highlighted in the literature: (i) collaborative filtering (new recommendations are based on the preferences of users similar to a target one) [4,28]; (ii) popularity-based recommendation (more popular content is delivered to users primarily) [29,30]; (iii) a target user’s preferences (new content should align with the focal user’s stance or their previous actions on the platform) [5]; and (iv) nudging (recommendations follow certain objectives of exogenous actors who govern the algorithm) [2].

### 2.3. Research on Ranking Algorithms

The literature on recommendation algorithms can be roughly divided into two core research directions, which are relatively loosely bound: (i) theoretical direction and (ii) empirical direction. In the first one, the emphasis is on elaborating new insights concerning ranking systems and their societal outcomes by means of agent-based modeling (despite the fact that some of these studies were informed by empirical data) [4,26,30,31]. The empirical direction focuses on discerning specific biases and disparities [3,32,33] in real-world personalization systems, as well as some temporal changes in their functioning [34]. For instance, scholars have found that ranking algorithms may display ideological biases and facilitate a particular side of the ideological spectrum [32]. However, these studies face many guardrails and confounders. Among others, it is extremely difficult to distinguish individual choices on a platform from the platform’s own mechanics [35,36,37].

Considering the modeling of recommendation systems, we would like to start our review with the study by Anderson et al. [31], who analyzed the specific mechanics of certain platforms called *badges*. Badges acknowledge users’ contributions to a platform (such as those applied on Stack Overflow) and thus serve as incentives. The goal of [31] was to understand how badges affect users’ behaviors. On top of that, ref. [31] posed a control problem in which one should allocate badges in an optimal fashion to modify users’ behaviors.

Next, in their seminal paper [4], Dandekar et al. studied the polarizing effect of several naive recommender systems in the presence of biased assimilation. This research question—how recommendation affects polarization and the formation of echo-chambers—was then extensively investigated in a large number of studies and received considerable attention in the literature [2,26,28,30,38,39,40,41,42,43,44]. Most of these studies relied on an agent-based approach [26,38,39], but some departed from agent-based models and subsequently moved to various forms of mean-field descriptions [30,42,43,45]. Many models [5,42,45,46] were built upon linear opinion formation protocols—such as the DeGroot and Friedkin–Johnsen models [10,14]. However, in [41], a nonlinear—bounded confidence—model was considered as a workhorse description of opinion evolution. On top of that, in [47], the authors applied the classical Voter model [48,49].

### 2.4. Investigating the Effect of Ranking Algorithms on Polarization

Mäs and Bischofberger [26] delved into whether personalization should facilitate polarization in social systems. They found that the answer to the question largely depends on the underlying opinion dynamics model. Geschke et al. [38] considered individual, social, and technological levels of information processing. They obtained that echo-chambers are inevitable outcomes of our cognitive mechanisms, with technological filtering amplifying these effects.

Perra and Rocha analyzed [2] how various features of social network structure affect opinion dynamics in the presence of different types of algorithmic ranking. They found that network topologies with high levels of clustering, or those with spatial correlations, as well as an absence of shortcuts, generally facilitate the formation of echo-chambers. The study by Peralta et al. [50] generally supported the findings of [2] on the role of modular networks in facilitating polarization for personalization regimes. They also found that, in the case of content filtering, pairwise social interactions promote polarization more than group-level interactions [51,52].

The research of Cinus et al. [39] stands out here as it concerns a link recommendation algorithm that suggests new contacts on a social media platform based on structural or vertex-based similarities. They found that this sort of recommendation can give rise to an increase in echo chambers, provided that an initial network is homophilic enough. A similar finding was obtained in [53], where the authors showed that linking structurally similar nodes amplifies opinion polarization due to the reinforcement of network modularity [54]. In fact, this is also consistent with the results of Perra et al. [2].

### 2.5. Ranking Algorithms and Optimization

Generally speaking, the purpose of ranking algorithms relates to maximizing some objective. Of course, we do not know exactly the organization of these algorithms and the composition of the underlying functionals, as they are a commercial secret. However, we can hypothesize that they maximize users’ engagement [5] or some other relevant metrics. In this vein, one can think of these algorithms as an optimization problem and thus apply optimization theory in their analyses. This approach was successfully implemented in [5], where Rossi et al. elaborated on a formal model, in which a user interacts with a news aggregator and changes their opinion in accordance with the Friedkin–Johnsen model [14]. While the user is inclined to prefer information that aligns with their current views (confirmation bias), the aggregator filters information, attempting to maximize the user’s engagement. The authors proved that this feedback loop displays a tendency towards users having more extreme opinions.

This model was then extended in [46] by considering a network of users communicating with each other and with a recommendation system. The authors conceptualized the work of the recommendation system by formulating a control problem, in which agents’ engagement over an infinite time horizon is maximized, both in model-dependent and model-independent scenarios.

However, the objective of a ranking algorithm may not relate to engagement maximization. For example, one can try to use this technology to affect individuals’ behaviors, as suggested in [31]. This formulation is extremely close to our current research. In the current paper, we consider a population of agents that communicate in accordance with an opinion formation model [7], and these communications are governed by a ranking algorithm. The algorithm is dynamically adjusted to affect agents’ opinions according to a predefined objective. Among other things, we consider a depolarization problem, in which an initially polarized society should be moderated.

Typically, scholars try to achieve such goals by solving an influence maximization problem [55], modifying the structure of the underlying network [56], or exposing agents to certain stimuli [57], which could be delivered to real users by bots or ads [8]. From this perspective, our approach to the problem is quite novel and has received relatively little attention in the literature.

## 3. Contributions

We depart from an opinion formation model that was first developed in [7] and then advanced in [8,58]. We have chosen this model because it provides a flexible framework to describe opinion evolution processes, as it is able to encode various forms of influence, such as assimilation, bounded confidence, or dissimilation [19]. For this model, we recall a mean-field approximation that takes the form of a nonlinear coarse-grained ordinary differential equation. In this equation, the state variables are the population-level parameters that represent the fractions of agents with a given opinion and a given type, while the ranking algorithm is operationalized as a set of time-dependent parameters that encode the probabilities that agents with given characteristics will be allowed to communicate with each other. The latter parameters appear linearly in the master equation.

We prove some properties of the mean-field description, such as the existence of a solution, its non-negativity, and continuation. Next, we set up a control problem in which the parameters of the ranking algorithm are dynamically adjusted to achieve a desirable opinion distribution, which is formalized by a linear objective functional.

We prove that this problem has a solution. Using the Pontryagin Maximum Principle, we derive some properties of optimal controllers. Applying finite-difference schemes, we solve the control problem for a simple scenario where the opinion alphabet consists of only two opinions—as in a two-party election. What is important is that the solution obtained in this case remains valid for any number of agent types and does not depend on external factors, such as the influence of social bots.

We perform extensive numerical tests to corroborate our findings. Comparing our controllers obtained through finite-difference schemes with open-loop controllers derived from numerical algorithms (the Forward–Backward Sweep method and the Direct method were applied as benchmarks), we conclude that the former performs as well as the latter.

Considering opinion spaces with more than two elements, we examine two generic scenarios: the depolarization of an initially polarized society and the nudging of a social system towards a given edge of an opinion spectrum. For these scenarios, we derive numerical solutions to the control problems and briefly discuss the resulting open-loop controllers.

## 4. Opinion Dynamics Model

### 4.1. Notations

By [m], we denote the set of natural numbers from 1 to m∈N, where N is the whole set of natural numbers. R stands for the set of real numbers. By $\delta_{i,j}$, we denote the Kronecker delta: $\delta_{i,j}=1$ if i=j and $\delta_{i,j}=0$ otherwise. Notation |A| refers to the cardinality of set A. We use both capital and lowercase letters to denote matrix objects. The inequality A≥0, where *A* is a matrix, indicates that all the components of *A* are non-negative.

In our derivations, we will typically consider systems of differential equations in matrix form. That is, instead of a vector of phase velocity, we will investigate a phase velocity matrix. This is due to the fact that our phase space will admit a natural separation into two macro-dimensions that stand for (i) opinions and (ii) types (see the model description below). And because of this, instead of using the conventional non-negative orthant $R_{+}^{n}$, we will harness the set $R_{+}^{m\times M}$ of all m×M matrices with non-negative components. Accordingly, $R_{-}^{m\times M}$ shows all m×M matrices with non-positive components. Analogously, the scalar product of two matrices *A* and *B* of the same shape m×M is defined as follows:

$$A\cdot B=\sum_{i\in [m]}\sum_{j\in [M]}A_{i,j}B_{i,j},$$

where $A_{i,j}$ and $B_{i,j}$ are the components of *A* and *B*, respectively.

It is worth noting that a differential equation in matrix form can be reshaped into a differential equation in vector form. As such, we can safely apply all the known theoretical results and constructions (for example, the Hamiltonian–Pontryagin formalism)—which imply vector representation—using matrix representation.

### 4.2. Agents and Their Attributes

Our workhorse opinion dynamics model was first presented in [7] and then elaborated on in [8,58]. In this model, *N* agents are immersed in a social network G=(V,E), where V={1,…,N} shows agents and E⊆V×V outlines edges between them. By $V_{i}$, we denote the neighbors of agent *i*. Each agent *i* is characterized by an opinion $x_{i}$ from set $Z=\{Z_{1},\ldots ,Z_{m}\}$ and a type $\xi_{i}$ from set $\Xi =\{\Xi_{1},\ldots ,\Xi_{M}\}$.

We assume that agents’ opinions can change, whereas their types are fixed. These types may stand for various human attributes, such as age, gender, education level, or combinations of these. For example, if we focus on two non-opinion characteristics—say age and education level—we can split the ranges of these two attributes into, say, $m_{1}$ and $m_{2}$ disjoint parts, respectively, and then derive $m_{1}\times m_{2}$ possible types. By introducing types into the model, we rely on the body of literature that suggests non-opinion characteristics, such as gender or age, affect how individuals influence their peers and, conversely, how open they are to the influence of their peers [59,60]. Besides sociodemographics, one can make use of psychological attributes—for instance, the Big Five psychometric traits or other relevant psychometric scales.

Next, each agent *i* is characterized by an activity parameter $\pi_{i}>0$, which shows how often the agent engages in conversations with their peers. This allows us to model heterogeneity in agents’ activity, which is frequently observed in real-world settings and may have a substantial effect on social dynamics [61]. For simplicity, we assume that activity parameters are functions of agents’ types: $\pi_{i}=\pi_{i}(\xi_{i})$.

### 4.3. Opinion Dynamics Protocol

In the model, agents communicate in consecutive pairwise interactions. In each time step t=1,2,…, an agent is randomly chosen as an influence object. This selection proceeds according to the activity distribution $\left\{\pi_{1},\ldots ,\pi_{N}\right\}$. Let us assume that an agent *i* has been picked. After that, an agent from $V_{i}$ is selected as an influence source. The probability that agent $j\in V_{i}$ will be chosen is given by $\frac{p_{j}}{\sum_{k\in V_{i}}p_{k}}.$ Let us assume that agent *j* has been selected.

After the agents *i* and *j* have been chosen, agent *i* is exposed to agent *j*’s opinion and has a chance to revise their current position. Let the opinions of *i* and *j* be $x_{i}(t)=Z_{s}$ and $x_{j}(t)=Z_{l}$, respectively. Let the agents’ types be $\xi_{i}=\Xi_{f}$ and $\xi_{j}=\Xi_{r}$, respectively. Now we are in a position to define how agent *i*’s opinion is revised. This revising procedure is derived from a Bernoulli trial with *m* possible outcomes $Z_{1},\ldots ,Z_{M}$ that come with the probabilities $p_{s,l,1}^{f,r},\ldots ,p_{s,l,m}^{f,r}$, where $p_{s,l,k}^{f,r}$ shows the likelihood that agent *i*’s new opinion will become $Z_{k}$.

It is worth emphasizing here that the upper indices of $p_{s,l,k}^{f,r}$ are synchronized with those of the communicating agents’ types, whereas the first two lower indices of $p_{s,l,k}^{f,r}$ stand for the agents’ prior opinions. The third index *k* in triplet s,l,k links to agent *i*’s potential opinion.

With these notations, $p_{s,l,s}^{f,r}=1$ indicates that agents with opinion $Z_{s}$ and type $\Xi_{f}$ do not change their opinions after being exposed to opinion $Z_{l}$ of an agent with type $\Xi_{r}$. To the contrary, $p_{s,l,s}^{f,r}=0$ means that in the same situation, the focal agent always modifies their opinion to $Z_{k}$. The quantities $p_{s,l,k}^{f,r}$ necessarily fulfill the normalization condition $p_{s,l,1}^{f,r}+\ldots +p_{s,l,m}^{f,r}=1.$

### 4.4. Transition Probability Tables

One can find it convenient to group quantities $p_{s,l,k}^{f,r}$ into a sequence of *transition probability tables*${\left\{P^{f,r}\right\}}_{f,r\in [M]}$ [7], where $P^{f,r}={\left(p_{s,l,k}^{f,r}\right)}_{s,l,k\in [m]}$. Each transition probability table, $P^{f,r}$, can be represented as a list of row-stochastic m×m matrices, $P_{1}^{f,r},\ldots ,P_{m}^{f,r}$, with $P_{s}^{f,r}={\left(p_{s,l,k}^{f,r}\right)}_{l,k\in [m]}$ showing how agents with opinion $Z_{s}$ and type $\Xi_{f}$ perceive influence from agents with type $\Xi_{r}$:

(1) $$P_{1}^{f,r}=\left(\begin{array}{ccc} p_{1,1,1}^{f,r} & \ldots & p_{1,1,m}^{f,r}\\ \ldots & \ldots & \ldots \\ p_{1,m,1}^{f,r} & \ldots & p_{1,m,m}^{f,r} \end{array}\right),\;\ldots ,\;P_{m}=\left(\begin{array}{ccc} p_{m,1,1}^{f,r} & \ldots & p_{m,1,m}^{f,r}\\ \ldots & \ldots & \ldots \\ p_{m,m,1}^{f,r} & \ldots & p_{m,m,m}^{f,r} \end{array}\right)$$

We will return to the discussion of transition probability tables formalism in the ongoing Section 4.7, where we will exemplify this approach of opinion dynamics representation and demonstrate its efficacy.

### 4.5. Adding Social Bots

In our model setup, we consider a scenario where the social system is augmented by agents of an extra-type $\Xi_{M+1}$. These agents are invulnerable to social influence and do not update their opinions. One can think of these agents as social bots or marketing messages that appear in users’ news feeds on social media. These agents may be controlled by one or more malicious actors. Below, we will refer to these agents as social bots, while other agents will be referred to as native or authentic ones.

In the current study, we are not interested in optimizing the behavior of social bots (this control problem was considered in [8], and we refer the interested Reader to this paper for more detail). We only say that the behavior of bots is defined exogenously by some person(s) and is known (strict assumption). By saying “behavior”, we mean the setup of opinions and targets of bots. We will clarify these issues in more detail below.

### 4.6. Personalization Algorithm

We assume that communications are curated by a specific algorithm that mimics artificial intelligence-based recommendation systems on real-world online social platforms. These systems aim to mitigate information overload, which is frequently faced by users [2]. Recommendation algorithms rely on specific information and metrics, including content popularity, users’ attributes, and users’ most recent actions [5]. Despite having a priori fair and unbiased targets, personalization systems are frequently accused of exacerbating individual information isolation and facilitating polarization in social communities [30]. Scholars argue that commercial companies, seeking to maximize the time users spend on social media sites, may adjust personalization algorithm metrics so that information communications may fall into the trap of popularity-biased and ideologically coherent interactions, with no access to less popular and challenging content [2,32].

We follow a rather simplified and interpretable approach wherein the personalization algorithm decides whether the two agents chosen for communication will actually communicate or not in a Bernoulli trial. Mathematically, the communication act between agents with opinions $Z_{s}$ and $Z_{l}$, and types $\Xi_{f}$ and $\Xi_{r}$, will proceed with a probability of $\Delta_{s,l}^{f,r}$. This operationalization allows us to consider various personalization strategies, including *homophily* (when agents with similar characteristics have a greater chance of interaction) and *heterophily* (when dissimilar agents communicate more often) with respect to both opinion and non-opinion attributes.

To summarize, the sequence of parameters $\left\{\Delta_{s,l}^{f,r}\right\}$, where s,l∈[m],f∈[M], and r∈[M+1] formalizes the personalization algorithm. If we fix opinion-related parameters *s* and *l* (the lower indices of $\Delta_{s,l}^{f,r}$), then we end up with M×(M+1) values that can be orchestrated into an M×(M+1) matrix, which shows the communication probabilities as functions of agents’ types. For example, the matrix

(2) $$\Delta_{s,l}=\left(\begin{array}{ccc} 1 & 0.75 & 0.5\\ 0.75 & 1 & 0.5 \end{array}\right)$$

for M=2 indicates that (i) native agents with similar types always communicate after being selected, (ii) native agents with different types communicate in 75 out of 100 cases, and (iii) communications between native agents and bots are allowed with a probability of 0.5 (for example, the platform itself may try to prevent ordinary users from spam attacks). Such a regime corresponds to homophily personalization.

Conversely, one can fix the type-related indices (the upper indices of $\Delta_{s,l}^{f,r}$) and examine how the opinions of interacting agents affect the probability that a communication will be allowed. In particular, for given f∈[M] and r∈[M+1], the matrix

(3) $$\Delta^{f,r}=\left(\begin{array}{ccc} 0.5 & 0.75 & 1\\ 0.75 & 0.5 & 0.75\\ 1 & 0.75 & 0.5 \end{array}\right)$$

(m=3) tells us that the personalization algorithm is biased towards facilitating communications between agents with opposite opinions—a so-called opinion–heterophily personalization strategy.

We would like to highlight that the personalization parameters—in contrast to the components of transition probability tables—are only constrained to lie in the interval [0;1] and do not follow any joint restrictions.

### 4.7. Interpreting Transition Probability Tables

In [8], it was systematically demonstrated how the transition probability table formalism may capture variant types of opinion formation mechanisms; therefore, we refer the interested Reader to [8] for a detailed inspection. For now, we will provide only a few examples to comprehend the organization of transition probability tables.

Let us start from a situation when there is only one agent type, so the upper indices of the transition probabilities can be safely omitted.

**Example** **1.** *Let* m=2 *—such a situation may occur, say, in a two-party election system. Let us consider the following transition probability table:*

(4) $$P_{1}=\left(\begin{array}{cc} 1 & 0\\ 0 & 1 \end{array}\right),P_{2}=\left(\begin{array}{cc} 1 & 0\\ 0 & 1 \end{array}\right).$$

*One can notice that the transition probability table, *(4)*, maps the dynamics of the Voter model [48,62] where agents simply copy the opinions of their conversation partners.*

However, as it was documented in the empirical studies [21,63,64], in real-world settings, individuals rarely change their opinions. Because of this, in any matrix $P_{k}$ in Formula (1), the *k*-th column—a column that comprises the probabilities of keeping the current opinion unchanged—should dominate. This is perfectly illustrated in the ongoing example.

**Example** **2.** *Now we consider a transition probability table that was derived from empirical data that were harvested from an online social network (see [7] for details):*

(5) $$P_{1}=\left(\begin{array}{cc} 0.975 & 0.025\\ 0.952 & 0.048 \end{array}\right),P_{2}=\left(\begin{array}{cc} 0.066 & 0.934\\ 0.049 & 0.951 \end{array}\right).$$

*The transition probability table, *(5)*, shows three important takeaways that were confirmed in various (both laboratory and field) studies on social influence: (i) an individual may change their opinion even after communicating with a confederate [63,64]; (ii) after being exposed to the opposite opinion, an individual tends to change their opinion more often than if being exposed to the same opinion [21]; (iii) opinion evolution patterns are usually asymmetric [65]. In our case, the last point means that individuals with opinions *$Z_{1}$* and *$Z_{2}$* react differently to the opposite opinions (mathematically, matrix *$P_{1}$* does not turn to *$P_{2}$* after flipping the rows and columns).*

If one considers opinion alphabets with more than two elements, then it becomes possible to capture more subtle forms of social influence. For example, the following transition probability table embraces a bounded confidence mechanism of opinion dynamics [19]:

**Example** **3.** *Let us consider the following transition probability table:*

(6) $$\begin{array}{c} P_{1}=\left(\begin{array}{ccc} 1 & 0 & 0\\ 0.8 & 0.2 & 0\\ 0.9 & 0.1 & 0 \end{array}\right),P_{2}=\left(\begin{array}{ccc} 0.25 & 0.7 & 0.05\\ 0 & 1 & 0\\ 0.05 & 0.7 & 0.25 \end{array}\right),P_{3}=\left(\begin{array}{ccc} 0 & 0.1 & 0.9\\ 0 & 0.2 & 0.8\\ 0 & 0 & 1 \end{array}\right). \end{array}$$

*For now, we are in a three-element ordinal opinion alphabet. By saying “ordinal”, we mean that opinions* $Z_{1}$ *,* $Z_{2}$ *, and* $Z_{3}$ *are arranged, with* $Z_{1}$ *and* $Z_{3}$ *representing opposite standpoints, while* $Z_{2}$ *shows a neutral stance. Having ordered opinions, we may appreciate assimilative opinion shifts (directed towards the opinion of an influence source) and dissimilative ones (directed outwards) [19]. (In our notations, assimilative opinion shifts are described by those components,* $p_{s,l,k}$ *, whose indices fulfill the equality* (k−s)×(l−s)>0 *; the inequality* (k−s)×(l−s)<0 *in turn marks a dissimilative opinion shifts).*

*From *(6)*, one can tell that if the opinion of an influence object is *$Z_{2}$*, then it changes to *$Z_{1}$* with a probability of 0.25 after communication with opinion *$Z_{1}$* (assimilative opinion shift) and with a probability of 0.05 after communication with opinion *$Z_{3}$* (dissimilative opinion shift). We would like to highlight that such patterns are not merely illustrative and imaginary, but were documented in empirical data [7].*

*Transition probability table *(6)* also demonstrates that agents with extreme opinions (*$Z_{1}$* or *$Z_{3}$*) can modify their opinions only to *$Z_{2}$*. On top of that, they do this more frequently when exposed to the neighboring opinion (*$Z_{2}$*) than when exposed to the opposite opinion (*$Z_{3}$* or *$Z_{1}$*, respectively)—a phenomenon that is usually referred to as bounded confidence [15,66].*

*Finally, *(6)* is perfectly symmetric, which ensures that opinions *$Z_{1}$* and *$Z_{3}$* have no edges over each other.*

If one wants to capture a scenario with more than one agent type and where individuals’ perceptions of influence change across in- and cross-type communications, then one should apply various transition probability tables depending on the types of the interacting agents. In particular, it stands to reason that for in-type communications, the level of conformity should be higher than that of cross-type communications—see, for example, Ref. [57]. This effect may be parameterized by increasing the probability of opinion assimilation at the expense of the probability of maintaining the current opinion for transition probability tables dedicated to in-type interactions. To be more specific, one can employ (6) to describe interactions between agents of different types, and the following transition probability table, which provides more room for conformity and assimilative shifts, can be employed in in-type communications:

(7) $$\begin{array}{c} P_{1}=\left(\begin{array}{ccc} 1 & 0 & 0\\ 0.7 & 0.3 & 0\\ 0.8 & 0.2 & 0 \end{array}\right),P_{2}=\left(\begin{array}{ccc} 0.35 & 0.6 & 0.05\\ 0 & 1 & 0\\ 0.05 & 0.6 & 0.35 \end{array}\right),P_{3}=\left(\begin{array}{ccc} 0 & 0.2 & 0.8\\ 0 & 0.3 & 0.7\\ 0 & 0 & 1 \end{array}\right). \end{array}$$

## 5. Mean-Field Approximation

### 5.1. Assumptions and Notations

In the interest of notations, in what follows, we will refer to agents’ activity rates via the lower indices of their types. So, if $\xi_{i}=\Xi_{f}$, then instead of $\pi_{i}$, we will simply write $\pi_{f}$.

We depart from the assumption that the underlying social network is initiated by a stochastic block model [67]. This family of network generation algorithms takes a set of nodes divided into disjoint subsets (blocks) and then creates ties between nodes in an independent fashion. For each pair of nodes, the probability of a tie appearing is a function of the blocks to which the nodes pertain. These probabilities are the parameters of the model. As a result, one is given the opportunity to flexibly adjust various in- and inter-block tie appearance rates depending on the modeler’s purposes.

Let us now define how ties between native agents form. We assume that blocks correspond to agents’ types. We have *M* types of authentic agents in total, so we end up with *M* blocks. We denote the size of block *f* with $N_{f}=\left\{i\in V\;|\;\xi_{i}=\Xi_{f}\right\}$. For each pair, f,r∈[M], of blocks, we introduce the quantity $\rho_{f,r}\in \left[0;1\right]$ that gives the probability that a randomly chosen pair of vertices of the corresponding types $\Xi_{f}$ and $\Xi_{r}$ will be connected. Note that f=r leads to an in-type creation rate, whereas f≠r shows an inter-type creation rate (because the network is undirected by default, we have $\rho_{f,r}=\rho_{r,f}$ in this case). Following up on the empirical observations from real-world social networks, one can safely assume that $\rho_{f,f}$ should be greater than $\rho_{f,r}$ for a fixed pair f,r such that f≠r—this phenomenon is usually referred to as the homophily or modularity of social networks [54].

Next, following the approach of [8], we assume that social bots act in a personalized fashion and can apply various manipulation strategies depending on the types of their targets. Bots are grouped into disjoint subsets (*cohorts*), and each cohort focuses on a specific type of authentic agent. In other words, the cohorts of bots and the corresponding blocks of native agents organize bipartite graphs. By $U_{f}(t)$, we denote the population of cohort *f* (which includes the bots that focus on the native agents of type $\Xi_{f}$), and by $\rho_{f}(t)\in \left[0;1\right]$, we denote the intensity of connections between the native agents of type $\Xi_{f}$ and the social bots from the corresponding cohort *f* at time *t*. The time argument indicates that social bots may act adaptively, so the compositions of the cohorts, as well as the communication intensity rates, may change over time. For example, at some moment, all bots may find themselves in one cohort—meaning that all the bots exert influence on only one type of native agent. These agents may be, for instance, the most prone to conformity and thus are most vulnerable to influence.

As a result, we end up with a dynamic stochastic block model with 2×M blocks of sizes $N_{1},\ldots ,N_{M},U_{1}(t),\ldots U_{m}(t)$ (some of them may be empty) and the following sequence of edge creation probabilities: ${\left\{\rho_{f,r}\right\}}_{f,r\in [M]},{\left\{\rho_{f}\left(t\right)\right\}}_{f\in [M]}.$ Note that for any *t*, it holds that

$$N_{1}+\ldots +N_{M}+U_{1}(t)+\ldots +U_{m}(t)=N.$$

**Figure 1:**
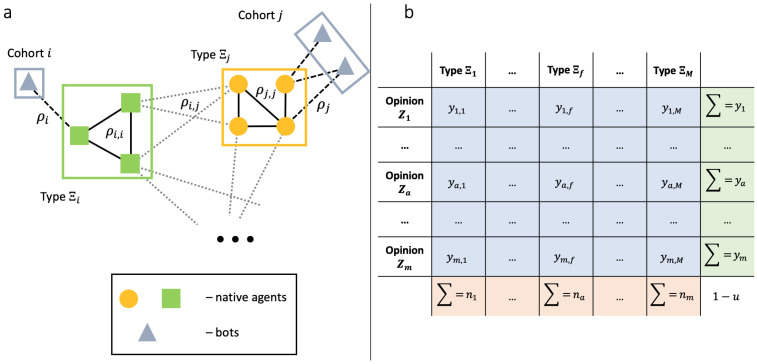
(**a**) We showcase a sketch of the network structure. The network consists of *M* blocks of native agents and *M* cohorts of bots, for a total of 2×M blocks. In the interest of plot, we show only 4 blocks. Ties between native agents derive from their types. It means that in-type ties (solid lines) may be overrepresented, compared to cross-type connections (dashed pale). Bots organize bipartite graphs with their targets (dashed lines). (**b**) Our state variable is $y_{a,f}$—the fraction of authentic agents with type $\Xi_{f}$ that have opinion $Z_{a}$ at a given time moment. If we sum this quantity over all possible opinions, then we end up with the number of agents of type $\Xi_{f}$—$n_{f}$. The summation over all possible types lead us to the number of agents with opinion $Z_{a}$, which is given by $y_{a}$. The total summation gives us the fraction of authentic agents y=1−u.

Figure 1a schematically illustrates our assumptions about the network structure. Once again, we assume that connections between native agents are static, but ties between native agents and bots, as well as the cohort populations, may change over time. We would like to highlight that, despite bots acting strategically, the appearance of ties between authentic agents and bots does not depend on the agents’ opinions—only on their types. In principle, this modification can be incorporated into the model, but the computations will become more cumbersome.

### 5.2. Introducing Population-Level Variables

Let us define some macroscopic variables that we will make use of in our derivations. First, by $y_{a,f}(t)$ (a∈[m],f∈[M]), we will denote the fraction of native agents having opinion $Z_{a}$ and type $\Xi_{f}$ at time *t*:

$$y_{a,f}(t)=\frac{\left|\{i\in V\;|\;x_{i}\left(t\right)=Z_{a},\xi_{i}=\Xi_{f}\}\right|}{N}.$$

Next, $n_{f}$ denotes the total fraction of agents with type $\Xi_{f}$, $n_{f}=\sum_{a\in [m]}y_{a,f}(t)$, and $y_{a}(t)$ denotes the total fraction of agents with opinion $Z_{a}$ at time *t*, $y_{a}=\sum_{f\in [M]}y_{a,f}(t)$. The quantity $u_{l,r}(t)$ shows the fraction of bots from cohort *r* with opinion $Z_{l}$ at time *t*, $u_{r}(t)$ represents the total fraction of bots in cohort *r*, and $u=u_{1}(t)+\ldots +u_{M}(t)$ indicates the total fraction of bots in the system. Apparently, we have

$$\underset{\mathrm{native}\;\mathrm{agents}}{\underbrace{n_{1}+\ldots +n_{m}}}+\underset{\mathrm{bots}}{\underbrace{u_{1}\left(t\right)+\ldots +u_{m}\left(t\right)}}=1.$$

This organization of our phase space is schematically illustrated in Figure 1b.

### 5.3. Master Equation

Having all these notations, we are now in a position to write the mean-field approximation. It was derived in [58], and we refer the interested Reader to this paper for detailed computations. Let τ be the scaled time: $\tau =\frac{t}{N},\delta \tau =\frac{1}{N}$. Let $\Omega_{f,r}=\pi_{r}\cdot \rho_{f,r},\Omega_{f}(\tau )=\pi_{M+1}\cdot \rho_{f}(\tau )$, and

$$A=\sum_{f\in [M]}n_{f}\cdot \pi_{f}+u\cdot \pi_{M+1},\;B_{f}(\tau )=\sum_{r\in [M]}n_{r}\cdot \Omega_{f,r}+u_{f}(\tau )\cdot \Omega_{f}(\tau ).$$

The following coarse-grained nonlinear ODE holds when applying the thermodynamic limit N→∞:

(8) $$\begin{array}{c} {\dot{y}}_{a,f}=\frac{\pi_{f}}{A\cdot B_{f}\left(\tau \right)}[\sum_{s,l\in [m]}y_{s,f}(\sum_{r\in [M]}y_{l,r}\cdot \Omega_{f,r}\cdot \Delta_{s,l}^{f,r}\left(\tau \right)\cdot p_{s,l,a}^{f,r}\\ +u_{l,f}\left(\tau \right)\cdot \Omega_{f}\left(\tau \right)\cdot \Delta_{s,l}^{f,M+1}\left(\tau \right)\cdot p_{s,l,a}^{f,M+1})\\ -y_{a,f}\sum_{l\in [m]}\left(\sum_{r\in [M]}y_{l,r}\cdot \Omega_{f,r}\cdot \Delta_{a,l}^{f,r}\left(\tau \right)+u_{l,f}\left(\tau \right)\cdot \Omega_{f}\left(\tau \right)\cdot \Delta_{a,l}^{f,M+1}\left(\tau \right)\right)],\\ a\in [m],f\in [M]. \end{array}$$

In the master Equation (8), quantities *A* and $B_{f}(\tau )$ serve as normalization values—they appear after computing the probabilities of picking up an agent with a particular opinion and a particular type as an influence object (*A*) and then selecting an agent with a particular opinion and a particular type as an influence source ($B_{f}$). In the latter case, a social bot can be chosen, and since the cohorts of bots change over time, the quantity $B_{f}(\tau )$ has a time argument.

**Remark** **1.** *Here, we would like to highlight that for Equation *(8)* to be correct, it is necessary to require that the opinions, cohorts, and ties change “no faster” than the speed at which the system evolves.*

It is worth noting that the right-hand part of (8) is quadratic with respect to the populations of native agents and bots. This is due to the fact that communication events in the model are essentially pairwise. In this respect, (8) differs from many compartmental (epidemiological) models [68,69,70,71,72], such as the SIR model, whereby linear terms are frequently encountered. Next, one may notice that (8) is homogeneous with respect to the tie densities $\rho_{f,r}$ and $\rho_{f}$: if introducing a simultaneous variable map $\rho_{f,r}\to \rho_{f,r}\times \rho$ and $\rho_{f}\to \rho_{f}\times \rho$, where ρ>0, then the equation remains the same.

As a final remark, we would also like to highlight that in (8), the parameters of the personalization algorithm are not static, but change over time.

### 5.4. Properties of the Master Equation

For ease of notation, we denote the right-hand side of (8) as $F_{a,f}$. Next, we make use of the matrix functions defined as follows:

$$y={\left(y_{a,f}\right)}_{a\in [m],f\in [M]},\;F={\left(F_{a,f}\right)}_{a\in [m],f\in [M]},$$

$$\Delta ={\left(\Delta_{s,l}^{f,r}\right)}_{s,l\in [m]}^{f\in [M],r\in [M+1]}.$$

Using these notations, we rewrite (8) as

(9) $$\dot{y}=F(\tau ,y,\Delta \left(\tau \right)).$$

Let the functions $\Omega_{f}(\tau )$ and $u_{l,f}(\tau )$ for l∈[m], f∈[M] be measurable. Let the personalization algorithm function Δ(τ) be measurable. It is not a difficult task to show that, in this case, the Cauchy problem for Equation (9) with the initial condition

(10) $$y_{a,f}(\tau_{0})=q_{a,f},\;a\in \left[m\right],f\in \left[M\right],$$

or, in matrix form

(11) $$y(\tau_{0})=q,$$

where the natural restrictions

(12) $$q_{a,f}\geq 0,\;\sum_{a\in [m]}q_{a,f}=n_{f}$$

on the initial point are fulfilled, has a unique solution y(τ) that is defined on some interval I⊂R. This solution y(τ) is an absolutely continuous function and satisfies (9) almost everywhere [73,74].

Our current purpose is to show that y(τ) is non-negative. In other words, we need to show that $R_{+}^{m\times M}$ is an invariant set for (9). To prove this, we shall take an arbitrary point *y* at the boundary of $R_{+}^{m\times M}$ and then check if the scalar product of the outer normal of $R_{+}^{m\times M}$ in *y* and the right-hand part of (9) is non-positive for any τ [75]. Let us assume that $y_{a,f}=0$ for $(a,f)\in I^{*}\subset \left[m\right]\times \left[M\right]$. For such pairs of *a* and *f*, we have

$$\begin{array}{c} F_{a,f}=\frac{\pi_{f}}{A\cdot B_{f}\left(\tau \right)}[\sum_{s,l\in [m]}y_{s,f}(\sum_{r\in [M]}y_{l,r}\cdot \Omega_{f,r}\cdot \Delta_{s,l}^{f,r}\left(\tau \right)\cdot p_{s,l,a}^{f,r}\\ +u_{l,f}\left(\tau \right)\cdot \Omega_{f}\left(\tau \right)\cdot \Delta_{s,l}^{f,M+1}\left(\tau \right)\cdot p_{s,l,a}^{f,M+1})]\geq 0. \end{array}$$

Next, under our assumptions, the set of the outer normals is given by $\nu ={\left(\begin{array}{c} \nu_{a,f} \end{array}\right)}_{a\in [m],f\in [M]}$, where $\nu_{a,f}=0$ if $(a,f)\notin I^{*}$ and $\nu_{a,f}$ is a negative value if $(a,f)\in I^{*}$. As such, the scalar product of ν and *F* would be less than or equal to zero. Thus far, we have obtained the following result.

**Statement** **1.** *Assume that the initial condition satisfies *q≥0*. Let *y(τ)* be the solution to the Cauchy problem in *(9) and (11)* on some interval I. Then *y(τ)* is non-negative on I.*

Next, one can straightforwardly notice that the functions $\sum_{a\in [m]}y_{s,f}$ for f∈[M] are the first integrals of (8), which reflects the fact that agents’ types remain unchanged. As such, given Statement 1, y(τ) is bounded from above, and the following result is true.

**Statement** **2.** *Assuming that the conditions in *(12)* are fulfilled. Then the solution *y(τ)* to the Cauchy problem in *(9)* and *(11)* can be extended on *R*.*

Having this, we can be sure that the model in (9) and (11) makes sense for the population densities $y_{s,f}$, and that the trajectories of the system can be extended to the right. Therefore, we can harness this model to formulate control problems.

### 5.5. Simulation Examples

Before moving on to the setup of a control problem, we provide some illustrative examples that showcase the dynamics of the model and the accuracy of the mean-field description. We consider the case m=3,M=2. We employ the transition probability tables in (6) and (7). The table in (6) covers interactions between agents of different types, while (7) describes in-type interactions. We assume that N=5000. Of these, $N_{1}=2500$ agents have type $\Xi_{1}$, $N_{2}=2000$ agents have type $\Xi_{2}$, and U=500 agents are bots. The initial joint distribution of opinions and types is given by

$$q=\left(\begin{array}{cc} 0.4 & 0\\ 0.1 & 0.1\\ 0 & 0.3 \end{array}\right).$$

This said, we consider a polarized society wherein the agents with type $\Xi_{1}$ are more inclined to opinion $Z_{1}$, and agents with type $\Xi_{2}$ prefer $Z_{3}$.

We assume that bots apply a constant strategy: they target the first-type agents and influence them with opinion $Z_{3}$ all the way. Mathematically, it means that $u_{3,1}(\tau )\equiv 0.1$. The stochastic block model parameters are as follows: $\rho_{1,1}=\rho_{2,2}=0.4$, $\rho_{1,2}=\rho_{2,1}=0.1$, and $\rho_{1}(\tau )\equiv 0.1$. The activity parameters are defined as follows: $\pi_{1}=2,\pi_{1}=1,$ and $\pi_{3}=3$. We assume that the first-type agents are more active than agents of type $\Xi_{2}$, but that the bots exhibit the highest activity rate, as is usually the case on social media platforms.

Three ranking algorithm specifications are considered. In the first one, no ranking is applied, and all the components of Δ are equal to one. The second specification refers to type homophily. For each s,l∈[m], matrix $\Delta_{s,l}(\tau )$ is defined by (2). Finally, the third specification facilitates opinion heterophily according to the matrix in (3).

**Figure 2:**
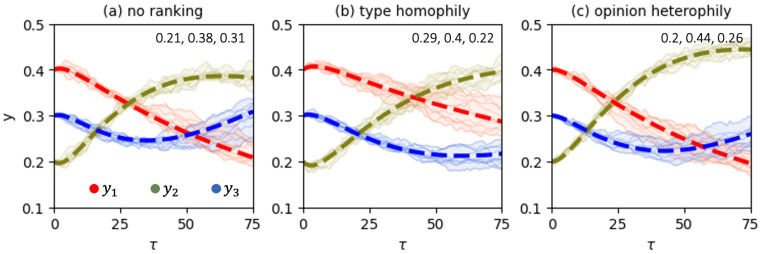
Comparing the direct simulations with the stochastic opinion dynamics models (pale lines, each line corresponds to one of 10 independent experiments) against the corresponding solutions of the mean-field equation (bold dashed lines). Each trajectory marks the total fraction of agents with the corresponding opinion irrespective of their types. The values of the order parameters at the terminal time τ=75 (that corresponds to T=5000×75= 375,000 model iterations) are plotted in the upper right corner of each panel.

Figure 2 shows direct simulations with the stochastic model and compares them against the solutions of the master equation, Equation (9). First, we notice that the mean-field description yields a reasonable level of accuracy in approximating the behavior of the stochastic system. We also appreciate the substantial effect of varying the ranking algorithm on opinion dynamics. One more interesting observation from Figure 2 is that in the absence of ranking, the social system features the highest level of polarization—the population $y_{2}$ of “neutral” agents reaches the smallest value of 0.38.

## 6. Control Problem

Let us assume that a person—say, an owner of a social media platform—also (in addition to the owner(s) of social bots) attempts to sway the native agents’ opinions. For instance, the platform owner may notice that the bots tear apart public opinion and polarize society. To this end, the owner may decide to apply a depolarization intervention in response. How can they do that? We assume that the platform owner is able to adjust the personalization algorithm. Mathematically, it means that the parameters $\Delta_{s,l}^{f,r}$ are control variables and are subject to variation.

With this in mind, we formulate the following control problem:

(13) $$\begin{array}{c} J⟶\min_{\Delta (.)},\\ \frac{dy}{d\tau }=F\left(\tau ,y,\Delta \left(\tau \right)\right),\;\tau \in \left[0;T\right],\\ y(0)=q,\\ \Delta (\tau )\in F,\;\tau \in [0;T], \end{array}$$

where *J* is the following linear objective:

(14) $$J=K\int_{0}^{T}\sum_{\alpha \in [m]}v_{\alpha }y_{\alpha }\left(\tau \right)d\tau +\sum_{\alpha \in [m]}v_{\alpha }y_{\alpha }\left(T\right).$$

In (14), the value of *T* outlines a time horizon. The weight vector $v=(v_{1},\ldots ,v_{m})$ represents the objective of the platform owner. A larger value of $v_{i}$ indicates that opinion $Z_{i}$ should be less represented in the population. As demonstrated in Ref. [8], because agents do not leave the system, we can safely assume that the weight vector is non-negative. Besides this, an algorithm for adjusting the values of the weight vector, given prior knowledge of the social system at hand, can also be found in [8]. Our final remark on (14) is that the parameter K>0 shows the relative importance of the integral term of the functional.

The class of admissible controllers F includes all measurable functions Δ(τ) such that

$$F=\{\Delta^{min}\leq \Delta_{s,l}^{f,r}\leq \Delta^{max},\;s,l\in \left[m\right],\;f\in \left[M\right],\;r\in \left[M+1\right]\}$$

on [0;T]. Parameter $\Delta^{min}$ represents a floor value for personalization. This implies that all agents, regardless of their types and opinions, should have some minimal chance to communicate. The setting $\Delta^{min}=0$ shows an extreme case in which the personalization algorithm can completely close communication channels. By default, we assume that $\Delta^{max}=1$.

## 7. Existence, Uniqueness, and Necessary Conditions for Optimality

We first reformulate our control problem by introducing one additional phase variable $y_{*}$, which is defined as follows:

$$y_{*}(\tau )=K\int_{0}^{\tau }\sum_{\alpha \in [m]}v_{\alpha }y_{\alpha }\left(\xi \right)d\xi .$$

As a result, we end up with the following Mayer problem:

(15) $$\begin{array}{c} y_{*}(T)+\sum_{\alpha \in [m]}v_{\alpha }y_{\alpha }\left(T\right)⟶\min_{\Delta (.)},\\ {\dot{y}}_{*}=K\sum_{\alpha \in [m]}v_{\alpha }y_{\alpha },\;\dot{y}=F\left(\tau ,y,\Delta (\tau )\right),\;\tau \in \left[0;T\right],\\ y(0)=q,\\ \Delta (\tau )\in F,\;\tau \in [0;T]. \end{array}$$

Control problem (15) has a convex compact set of admissible controls. Next, the control process of (15) is linear in control. This guarantees that for each $\left(\tau ,y,y_{*}\right)$, the set $\left\{(K\sum_{\alpha \in [m]}v_{\alpha }y_{\alpha },F(\tau ,y,\Delta ))\;|\;\Delta \in F\right\}$ is compact and convex. As such, due to Filippov’s theorem, the reachable set is also compact. Because the cost functional in (15) includes only the terminal term, which is a continuous function depending solely on the phase variables, we arrive at the following result.

**Statement** **3.** *The solution to the control problem in *(13)* exists.*

**Remark** **2.** *The uniqueness of this solution is not necessarily fulfilled. For example, there could be more than one controller that yields* J=0 *for control problems posed for “well-controlled” systems with* k=0 *.*

Let us now write the Hamiltonian–Pontryagin function for the control problem in (13) (here, we account for the fact that the value of y(T) is not fixed):

$$\begin{array}{c} H\left(\tau ,y,\Lambda ,\lambda \left(\tau \right)\right)=\sum_{a\in [m]}\sum_{f\in [M]}\left[-K\cdot v_{a}\cdot y_{a,f}+\lambda_{a,f}\left(\tau \right)F_{a,f}\left(\tau ,y,\Lambda \right)\right]. \end{array}$$

From the Pontryagin Maximum Principle, we know that if a pair $\hat{\Lambda }\left(\tau \right),\hat{y}\left(\tau \right)$ is optimal, then there exists a function $\lambda \left(\tau \right)={\left(\begin{array}{c} \lambda_{i,j}\left(\tau \right) \end{array}\right)}_{i\in [m],j\in [M]},$ that is the solution to the Cauchy problem:

(16) $$\begin{array}{c} \frac{d\lambda_{i,j}}{d\tau }=Kv_{i}-\sum_{a\in [m]}\sum_{f\in [M]}\lambda_{a,f}\frac{\partial F_{a,f}\left(\tau ,\hat{y}\left(\tau \right),\hat{\Lambda }\left(\tau \right)\right)}{\partial y_{i,j}},\\ \lambda_{i,j}\left(T\right)=-v_{i}, \end{array}$$

and we have almost everywhere that

$$\max_{\Delta (\tau )\in F}H\left(\tau ,\hat{y}(\tau ),\Lambda (\tau ),\lambda (\tau )\right)=H\left(\tau ,\hat{y}(\tau ),\hat{\Lambda }(\tau ),\lambda (\tau )\right).$$

Since the Hamiltonian–Pontryagin function is linear with respect to the control variables, we can try to understand the organization of the optimal control in some specific cases by eliciting switching functions [71].

**Statement** **4.** *Let *λ(τ)* be the solution to the Cauchy problem *(16)* for an optimal pair *$\hat{\Lambda }\left(\tau \right),\hat{y}\left(\tau \right)$*. Let us consider the quantity*

(17) $$Q_{i,j}^{f,k}\left(\tau \right)=\sum_{a\in [m]}\lambda_{a,f}\left(\tau \right)p_{i,j,a}^{f,k}-\lambda_{i,f}\left(\tau \right)$$

*for *i,j∈[m]* and *f∈[M],k∈[M+1]*, and *τ∈[0;T]*. Let *${\hat{y}}_{i,f}\left(\tau \right)\neq 0$* and either *${\hat{y}}_{j,k}\left(\tau \right)\neq 0,\Omega_{f,k}\neq 0$* (if *k≤M*), or *$u_{j,f}\left(\tau \right)\neq 0$* and *$\Omega_{f}\left(\tau \right)\neq 0$* (if *k=M+1*). Then we have*

(18) $$\begin{array}{c} {\hat{\Delta }}_{i,j}^{f,k}\left(\tau \right)=\left\{\begin{array}{c} \Delta^{max},\;\mathrm{if}\;\;Q_{i,j}^{f,k}\left(\tau \right)>0,\\ \Delta^{min},\;\mathrm{if}\;\;Q_{i,j}^{f,k}\left(\tau \right)<0. \end{array}\right. \end{array}$$

**Proof.**  Let us first calculate the derivatives of $F_{a,f}$ with respect to the control variables:

$$\begin{array}{c} \frac{\partial F_{a,f}}{\partial \Delta_{i,j}^{f,k}}=\frac{\pi_{f}}{A\cdot B_{f}}\left[y_{i,f}\cdot y_{j,k}\cdot \Omega_{f,k}\left(p_{i,j,a}^{f,k}-\delta_{i,a}\right)\right] \end{array}$$

for k∈[M], and

$$\begin{array}{c} \frac{\partial F_{a,f}}{\partial \Delta_{i,j}^{f,M+1}}=\frac{\pi_{f}}{A\cdot B_{f}}\left[y_{i,f}\cdot u_{j,f}\cdot \Omega_{f}\left(p_{i,j,a}^{f,M+1}-\delta_{i,a}\right)\right] \end{array}$$

for k=M+1.

After that, we substitute these derivatives into the expressions for $\frac{\partial H}{\partial \Delta_{i,j}^{f,k}}$ and $\frac{\partial H}{\partial \Delta_{i,j}^{f,M+1}}$ and obtain:

(19) $$\begin{array}{c} \frac{\partial H}{\partial \Delta_{i,j}^{f,k}}=\frac{\pi_{f}}{A\cdot B_{f}}y_{i,f}\cdot y_{j,k}\cdot \Omega_{f,k}\cdot Q_{i,j}^{f,k}, \end{array}$$

(20) $$\begin{array}{c} \frac{\partial H}{\partial \Delta_{i,j}^{f,M+1}}=\frac{\pi_{f}}{A\cdot B_{f}}y_{i,f}\cdot u_{j,f}\cdot \Omega_{f}\cdot Q_{i,j}^{f,k}. \end{array}$$

The statement to be proved directly follows from the expressions in (19) and (20). □

It is worth noting that Statement 4 is not informed regarding the value of ${\hat{\Lambda }}_{i,j}^{f,k}$ if $p_{i,j,i}^{f,k}=1$. This is intuitively clear: $p_{i,j,i}^{f,k}=1$ indicates that agents with opinion $Z_{i}$ and type $\Xi_{f}$ are not sensitive to influence from agents with opinion $Z_{j}$ and type $\Xi_{k}$. As such, these communications do not affect the state variable *y* and thus leave us in the dark as to how to accommodate them in optimal control finding.

These shortcomings fall under the more general umbrella of what one should do in the case when the derivatives of (19) and (20) are equal to zero on some interval $\hat{I}\subset R$. This could be the case because our Hamiltonian–Pontryagin function is linear with respect to Δ—otherwise, one would try to estimate the control variables from the resulting equations [72]. In this case—referred to as singular control [76]—one can calculate the full derivatives from (19) or (20) with respect to τ on $\hat{I}$ (in (21), we omit the arguments in the interest of space):

(21) $$\begin{array}{c} \frac{d}{d\tau }\frac{\partial H}{\partial \Delta_{i,j}^{f,k}}=\frac{\pi_{f}\cdot \Omega_{f,k}}{A\cdot B_{f}}\left[Q_{i,j}^{f,k}\left({\dot{y}}_{i,f}\cdot y_{j,k}+y_{i,f}\cdot {\dot{y}}_{j,k}\right)+y_{i,f}\cdot y_{j,k}\cdot {\dot{Q}}_{i,j}^{f,k}\right]\\ =\frac{\pi_{f}\cdot \Omega_{f,k}}{A\cdot B_{f}}[Q_{i,j}^{f,k}\left({\dot{y}}_{i,f}\cdot y_{j,k}+y_{i,f}\cdot {\dot{y}}_{j,k}\right)+y_{i,f}\cdot y_{j,k}\\ \times \left(\sum_{a\in [m]}{\dot{\lambda }}_{a,f}\cdot p_{i,j,a}^{f,k}-{\dot{\lambda }}_{i,f}\right)]=0. \end{array}$$

Note that the first equality in Equation (21) assumes that $B_{f}$ does not depend on τ. This is the case when the strategy of bots is constant over time.

After that, one should plug the expressions for $\dot{y}$ and $\dot{\lambda }$ into (21). This will give us an equation that is linear in the control variables, allowing us to find all the singular control components of Λ on $\hat{I}$. The resulting equation appears to be a bit too cumbersome, so we omit it in the interest of space. We make use of these calculations in the numerical solving of problem (13)—when applying the Forward–Backward Sweep method and maximizing the Hamiltonian–Pontryagin function pointwise (Our analysis showed that the use of singular control facilitates convergence of the FBS method. However, in most situations, it leads to boundary-bang controllers). Note that Statement 4 also informs our numerical algorithm.

We now turn to solving the target control problem using the finite-difference method.

## 8. Applying Finite-Difference Schemes: The Simplest Scenario M=2,M=1

In this section, we consider the case when there are only two opinions in the system, and only one (authentic) agent type exists: m=2,M=1. As such, we can safely omit all the indices that stand for agent types and bots cohorts. All we need is to separate the parameters of native agents from those of bots. We do this by applying the index “*u*”. As a result, we have the following master equation:

(22) $$\begin{array}{c} {\dot{y}}_{1}=\frac{\pi }{A\cdot B(\tau )}[\sum_{s=1}^{2}\sum_{l=1}^{2}y_{s}(y_{l}\cdot \Omega \cdot \Delta_{s,l}\left(\tau \right)\cdot p_{s,l,1}\\ +u_{l}\left(\tau \right)\cdot \Omega_{u}\left(\tau \right)\cdot \Delta_{s,l}^{u}\left(\tau \right)\cdot p_{s,l,1}^{u})\\ -y_{1}\left(\sum_{l=1}^{2}y_{l}\cdot \Omega \cdot \Delta_{1,l}\left(\tau \right)+\sum_{l=1}^{2}u_{l}\left(\tau \right)\cdot \Omega_{u}\left(\tau \right)\cdot \Delta_{1,l}^{u}\left(\tau \right)\right)]. \end{array}$$

Because $y_{1}+y_{2}=1$, Equation (22) describes the system completely.

Further, our control variables are given by

$$\Delta \left(\tau \right)=\left(\begin{array}{cc} \Delta_{1,1}\left(\tau \right) & \Delta_{1,2}\left(\tau \right)\\ \Delta_{2,1}\left(\tau \right) & \Delta_{2,2}\left(\tau \right) \end{array}\right),\;\Delta^{u}\left(\tau \right)=\left(\begin{array}{cc} \Delta_{1,1}^{u}\left(\tau \right) & \Delta_{1,2}^{u}\left(\tau \right)\\ \Delta_{2,1}^{u}\left(\tau \right) & \Delta_{2,2}^{u}\left(\tau \right) \end{array}\right).$$

Finally, we have the following cost functional:

$$J=K\int_{0}^{T}(v_{1}y_{1}(\tau )+v_{2}y_{2}(\tau ))d\tau +(v_{1}y_{1}(T)+v_{2}y_{2}(T)),$$

where v=(0,1) or v=(1,0). We can safely focus on these two weight vectors because all other weight configurations can be reduced to one of these two. Indeed, let us consider a weight vector $\left(v_{1},v_{2}\right)$, where $v_{2}>v_{1}$. As such, we can write $v_{2}=v_{1}+v^{\prime }$, with $v^{\prime }>0$. This leads us to

$$\begin{array}{c} J=K\int_{0}^{T}(v_{1}y_{1}\left(\tau \right)+\left(v_{1}+v^{\prime }\right))y_{2}\left(\tau \right))d\tau +(v_{1}y_{1}\left(T\right)+\left(v_{1}+v^{\prime }\right))y_{2}\left(T\right))\\ =-K\int_{0}^{T}v^{\prime }y_{1}\left(\tau \right)d\tau -v^{\prime }y_{1}\left(T\right)+KT\left(v_{1}+v^{\prime }\right)y+\left(v_{1}+v^{\prime }\right)y\\ ∼K\int_{0}^{T}y_{2}\left(\tau \right)d\tau +y_{2}\left(T\right), \end{array}$$

which means that from the perspective of the control problem at stake, a vector $\left(v_{1},v_{2}\right)$ with $v_{1}<v_{2}$ is equivalent to (0,1). Analogously, the case $v_{1}>v_{2}$ can be boiled down to (1,0).

Let us focus on the case v=(0,1)—that is, we want to decimate opinion $Z_{2}$ in the system. The opposite case can be elaborated on analogously. Hence, we have

$$J=K\int_{0}^{T}y_{2}\left(\tau \right)d\tau +y_{2}\left(T\right)$$

We approximate *J* using the trapezoidal rule:

$$\begin{array}{c} J=K\sum_{i=0}^{\frac{T}{\varepsilon }-1}\int_{i\varepsilon }^{(i+1)\varepsilon }y_{2}\left(\tau \right)d\tau +y_{2}\left(T\right)=K\sum_{i=0}^{\frac{T}{\varepsilon }-1}\frac{y_{2}\left(i\varepsilon \right)+y_{2}\left(\left(i+1\right)\varepsilon \right)}{2}\varepsilon +y_{2}\left(T\right)\\ =\frac{K\varepsilon }{2}y_{2}\left(0\right)+\varepsilon \sum_{i=0}^{\frac{T}{\varepsilon }-1}y_{2}\left(i\varepsilon \right)+\left(\frac{K\varepsilon }{2}+1\right)y_{2}\left(T\right). \end{array}$$

where ε>0 is small.

Considering $y_{1}+y_{2}=y$, the minimization of *J* can be replaced by the maximization of

(23) $$J^{\prime }=\varepsilon \sum_{i=0}^{\frac{T}{\varepsilon }-1}y_{1}\left(i\varepsilon \right)+\left(\frac{K\varepsilon }{2}+1\right)y_{1}\left(T\right).$$

We now approximate Equation (22) using the Euler scheme:

(24) $$\begin{array}{c} \frac{y_{1}\left(\left(i+1\right)\varepsilon \right)-y_{1}\left(i\varepsilon \right)}{\varepsilon }=\frac{\pi }{A\cdot B(i\varepsilon )}\\ \times [\sum_{s=1}^{2}\sum_{l=1}^{2}y_{s}\left(i\varepsilon \right)\cdot (y_{l}\left(i\varepsilon \right)\cdot \Omega \cdot \Delta_{s,l}\left(i\varepsilon \right)\cdot p_{s,l,1}\\ +u_{l}\left(i\varepsilon \right)\cdot \Omega_{u}\left(i\varepsilon \right)\cdot \Delta_{s,l}^{u}\left(i\varepsilon \right)\cdot p_{s,l,1}^{u})\\ -y_{1}\left(i\varepsilon \right)\cdot \left(\sum_{l=1}^{2}y_{l}\left(i\varepsilon \right)\cdot \Omega \cdot \Delta_{1,l}\left(i\varepsilon \right)+\sum_{l=1}^{2}u_{l}\left(i\varepsilon \right)\cdot \Omega_{u}\left(i\varepsilon \right)\cdot \Delta_{1,l}^{u}\left(i\varepsilon \right)\right)]. \end{array}$$

We consider a finite-difference analogue of the control problem in (13) with the discrete process, (24), and the cost functional, (23), to be maximized:

(25) $$\begin{array}{c} \varepsilon \sum_{i=0}^{\frac{T}{\varepsilon }-1}y_{1}\left(i\varepsilon \right)+\left(\frac{K\varepsilon }{2}+1\right)y_{1}\left(T\right)⟶\max_{\Delta (.)},\\ \mathrm{Process}\;(24),\;i=0,1,\ldots ,\frac{T}{\varepsilon }-1,\\ y(0)=q,\\ \Delta \left(i\varepsilon \right)\in F,\;i=0,1,\ldots ,\frac{T}{\varepsilon }-1. \end{array}$$

We assert the following result.

**Theorem** **1.** *The controller*

(26) $$\begin{array}{c} \Delta \equiv \left(\begin{array}{cc} \Delta^{min} & \Delta^{min}\\ \Delta^{max} & \Delta^{max} \end{array}\right),\;\Delta^{u}\equiv \left(\begin{array}{cc} \Delta^{min} & \Delta^{min}\\ \Delta^{max} & \Delta^{max} \end{array}\right) \end{array}$$

*is a solution of the control problem in *(25)*.*

**Proof.**  We first rewrite (24) as follows:

$$\begin{array}{c} y_{1}\left(\left(i+1\right)\varepsilon \right)=y_{1}\left(i\varepsilon \right)+\frac{\pi \cdot \varepsilon }{A\cdot B}\left[\left(A_{1}+A_{2}+A_{3}+A_{4}\right)+\left(A_{5}+A_{6}+A_{7}+A_{8}\right)\right], \end{array}$$

where

$$\begin{array}{c} A_{1}=y_{1}^{2}\left(i\varepsilon \right)\cdot \Omega \cdot \Delta_{1,1}\left(i\varepsilon \right)\cdot \left(p_{1,1,1}-1\right)\leq 0,\\ A_{2}=y_{1}\left(i\varepsilon \right)\cdot \left(y-y_{1}\left(i\varepsilon \right)\right)\cdot \Omega \cdot \Delta_{1,2}\left(i\varepsilon \right)\cdot \left(p_{1,2,1}-1\right)\leq 0,\\ A_{3}=y_{1}\left(i\varepsilon \right)\cdot \left(u-u_{1}\left(i\varepsilon \right)\right)\cdot \Omega_{u}\left(i\varepsilon \right)\cdot \Delta_{1,2}^{u}\left(i\varepsilon \right)\cdot \left(p_{1,2,1}^{u}-1\right)\leq 0,\\ A_{4}=y_{1}\left(i\varepsilon \right)\cdot u_{1}\left(i\varepsilon \right)\cdot \Omega_{u}\left(i\varepsilon \right)\cdot \Delta_{1,1}^{u}\left(i\varepsilon \right)\cdot \left(p_{1,1,1}^{u}-1\right)\leq 0,\\ A_{5}=\left(y-y_{1}\left(i\varepsilon \right)\right)\cdot y_{1}\left(i\varepsilon \right)\cdot \Omega \cdot \Delta_{2,1}\left(i\varepsilon \right)\cdot p_{2,1,1}\geq 0,\\ A_{6}={\left(y-y_{1}\left(i\varepsilon \right)\right)}^{2}\cdot \Omega \cdot \Delta_{2,2}\left(i\varepsilon \right)\cdot p_{2,2,1}\geq 0,\\ A_{7}=\left(y-y_{1}\left(i\varepsilon \right)\right)\cdot \left(u-u_{1}\left(i\varepsilon \right)\right)\cdot \Omega_{u}\left(i\varepsilon \right)\cdot \Delta_{2,2}^{u}\left(i\varepsilon \right)\cdot p_{2,2,1}^{u}\geq 0,\\ A_{8}=\left(y-y_{1}\left(i\varepsilon \right)\right)\cdot u_{1}\left(i\varepsilon \right)\cdot \Omega_{u}\left(i\varepsilon \right)\cdot \Delta_{2,1}^{u}\left(i\varepsilon \right)\cdot p_{2,1,1}^{u}\geq 0. \end{array}$$

One can notice from the above expressions that each quantity $A_{i}$ contains exactly one component of the controller. And, conversely, this very component appears only in $A_{i}$ and nowhere else.

Let us consider $i=\frac{T}{\varepsilon }-1$—this corresponds to the last moment T−ε before the terminal time comes. With that being said, to maximize $y_{1}(T)$ (and, correspondingly, to maximize the objective functional of (25)), the components of Δ(T−ε) that appear in non-negative terms ($A_{5},A_{6},A_{7},A_{8}$) should be set to the highest value possible ($\Delta^{max}$), and the components of Δ(T−ε) that appear in non-positive terms ($A_{1},A_{2},A_{3},A_{4}$) should be set to the minimal value possible ($\Delta^{min}$). As such, the following setup would be optimal if we were to start from $i=\frac{T}{\varepsilon }-1$:

$$\Delta (T-\varepsilon )=\left(\begin{array}{cc} \Delta^{min} & \Delta^{min}\\ \Delta^{max} & \Delta^{max} \end{array}\right),\;\Delta^{u}(T-\varepsilon )=\left(\begin{array}{cc} \Delta^{min} & \Delta^{min}\\ \Delta^{max} & \Delta^{max} \end{array}\right).$$

Continuing this reasoning and moving backward, we notice that for an arbitrary $i\in \{0,1,\ldots ,\frac{T}{\varepsilon }-1\}$, the control

$$\Delta (i\varepsilon )=\left(\begin{array}{cc} \Delta^{min} & \Delta^{min}\\ \Delta^{max} & \Delta^{max} \end{array}\right),\;\Delta^{u}(i\varepsilon )=\left(\begin{array}{cc} \Delta^{min} & \Delta^{min}\\ \Delta^{max} & \Delta^{max} \end{array}\right).$$

maximizes the value of $y_{1}(\left(i+1\right)\varepsilon )$. With this in mind, and applying the Bellman principle of optimality, we end up with the controller in (26). □

**Remark** **3.** *Intuitively, the controller in *(26)* is quite meaningful: if there are only two possible opinions, then it stands to reason that one should keep the agents holding a “desirable” opinion away from any contacts to prevent them from having any possibility of changing their opinion. And, on the contrary, one should facilitate communications between individuals with an “undesirable” opinion—to maximize the likelihood of opinion updating among these agents.*

**Remark** **4.** *One more interesting observation from the controller in *(26)* is that it does not depend on how the bots behave at all.*

## 9. Applying Finite-Difference Schemes: The Scenario M=2, M Is Arbitrary

Now we turn to a more meaningful scenario in which the number of agent types is arbitrary, but the opinion alphabet still includes only two elements. The fact that the control parameters appear independently in the process enables us to generalize our findings from the previous section. Applying the Euler approximation scheme again and employing the trapezoidal rule, thus boiling down the continuous control problem (13) to its discrete counterpart, results in a controller that is similar to that of (25).

For an arbitrary M≥1, we have the following cost functional:

$$J=K\int_{0}^{T}\sum_{r=1}^{M}(v_{1}y_{1,r}(\tau )+v_{2}y_{2,r}(\tau ))d\tau +\left(\sum_{r=1}^{M}v_{1}y_{1,r}(T)+v_{2}y_{2,r}(T)\right),$$

where $v=(v_{1},v_{2})$ is a weight vector. Without loss of generality, we consider $v_{1}=0$, $v_{2}=1$ (the number of opinion $Z_{2}$ holders is subject to minimization). We approximate the cost functional as follows:

$$\begin{array}{c} J=K\sum_{i=0}^{\frac{T}{\varepsilon }-1}\int_{i\varepsilon }^{(i+1)\varepsilon }\sum_{r=1}^{M}y_{2,r}\left(\tau \right)d\tau +\sum_{r=1}^{M}y_{2,r}\left(T\right)\\ =\sum_{r=1}^{M}\left[\frac{K\varepsilon }{2}y_{2,r}\left(0\right)+\varepsilon \sum_{i=0}^{\frac{T}{\varepsilon }-1}y_{2,r}\left(i\varepsilon \right)+\left(\frac{K\varepsilon }{2}+1\right)y_{2,r}\left(T\right)\right], \end{array}$$

where ε>0 is small.

For the sake of convenience, instead of minimizing *J*, we will maximize $J^{\prime }$:

(27) $$\begin{array}{c} J^{\prime }=\sum_{r=1}^{M}\left[\frac{K\varepsilon }{2}y_{1,r}\left(0\right)+\varepsilon \sum_{i=0}^{\frac{T}{\varepsilon }-1}y_{1,r}\left(i\varepsilon \right)+\left(\frac{K\varepsilon }{2}+1\right)y_{1,r}\left(T\right)\right]. \end{array}$$

Let f∈[M]. We now consider the differential equation for $y_{1,f}$:

(28) $$\begin{array}{c} {\dot{y}}_{1,f}=\frac{1}{A\cdot B_{f}\left(\tau \right)}\left[\sum_{s=1}^{2}\sum_{l=1}^{2}y_{s,f}\cdot \pi_{f}\cdot C_{s,l,1}^{f}\left(\tau \right)-y_{1,f}\cdot D_{1}^{f}\left(\tau \right)\right]\\ =\frac{1}{A\cdot B_{f}\left(\tau \right)}[y_{1,f}\cdot \pi_{f}\cdot C_{1,1,1}^{f}\left(\tau \right)+y_{1,f}\cdot \pi_{f}\cdot C_{1,2,1}^{f}\left(\tau \right)+y_{2,f}\cdot \pi_{f}\cdot C_{2,1,1}^{f}\left(\tau \right)\\ +y_{2,f}\cdot \pi_{f}\cdot C_{2,2,1}^{f}\left(\tau \right)-y_{1,f}\cdot D_{1}^{f}\left(\tau \right)], \end{array}$$

where

$$A=\sum_{i=1}^{M}n_{i}\cdot \pi_{i}+\sum_{j=1}^{M}u_{j}\cdot \pi_{M+1},\;B_{f}\left(\tau \right)=\sum_{r=1}^{M}n_{r}\cdot \Omega_{f,r}+u_{f}\cdot \Omega_{f,M+1},$$

$$C_{s,l,k}^{f}\left(\tau \right)=\sum_{r=1}^{M}y_{l,r}\cdot \Omega_{f,r}\cdot \Delta_{s,l}^{f,r}\left(\tau \right)\cdot p_{s,l,k}^{f,r}+u_{l,f}\left(\tau \right)\cdot \Omega_{f,M+1}\left(\tau \right)\cdot \Delta_{s,l}^{f,M+1}\cdot p_{s,l,k}^{f,M+1},$$

$$D_{1}^{f}\left(\tau \right)=\sum_{s=1}^{2}\sum_{r=1}^{M}y_{s,r}\cdot \Omega_{f,r}\cdot \Delta_{1,s}^{f,r}+\sum_{s=1}^{2}u_{s,f}\left(\tau \right)\cdot \Omega_{f,M+1}\left(\tau \right)\cdot \Delta_{1,s}^{f,M+1}.$$

Applying the Euler approximation scheme to (28), one ends up with:

(29) $$\begin{array}{c} \frac{y_{1,f}\left(\left(i+1\right)\varepsilon \right)-y_{1,f}\left(i\varepsilon \right)}{\varepsilon }=\frac{1}{A\cdot B_{f}\left(i\varepsilon \right)}[y_{1,f}\left(i\varepsilon \right)\cdot \pi_{f}\cdot C_{1,1,1}^{f}\left(i\varepsilon \right)\\ +y_{1,f}\left(i\varepsilon \right)\cdot \pi_{f}\cdot C_{1,2,1}^{f}\left(i\varepsilon \right)+y_{2,f}\left(\tau \right)\cdot \pi_{f}\cdot C_{2,1,1}^{f}\left(i\varepsilon \right)\\ +y_{2,f}\left(i\varepsilon \right)\cdot \pi_{f}\cdot C_{2,2,1}^{f}\left(i\varepsilon \right)-y_{1,f}\cdot D_{1}^{f}\left(i\varepsilon \right)]. \end{array}$$

Let us consider a finite-difference counterpart of the problem in (13) with the discrete process, (29), and the cost functional, (27), to be maximized:

(30) $$\begin{array}{c} \sum_{r=1}^{M}\left[\frac{K\varepsilon }{2}y_{1,r}\left(0\right)+\varepsilon \sum_{i=0}^{\frac{T}{\varepsilon }-1}y_{1,r}\left(i\varepsilon \right)+\left(\frac{K\varepsilon }{2}+1\right)y_{1,r}\left(T\right)\right]⟶\max_{\Delta (.)},\\ \mathrm{Process}\;(29),\;i=0,1,\ldots ,\frac{T}{\varepsilon }-1,\\ y(0)=q,\\ \Delta \left(i\varepsilon \right)\in F,\;i=0,1,\ldots ,\frac{T}{\varepsilon }-1. \end{array}$$

We assert the following result.

**Theorem** **2.** *The controller*

(31) $$\begin{array}{c} \Delta^{f,r}\equiv \left(\begin{array}{cc} \Delta^{min} & \Delta^{min}\\ \Delta^{max} & \Delta^{max} \end{array}\right),\;\Delta^{f,M+1}\equiv \left(\begin{array}{cc} \Delta^{min} & \Delta^{min}\\ \Delta^{max} & \Delta^{max} \end{array}\right) \end{array}$$

* is a solution to the control problem in *(30)*.*

**Proof.**  After rearranging the terms on the right-hand side of (29), we have

$$\begin{array}{c} y_{1,f}\left(\left(i+1\right)\varepsilon \right)=y_{1,f}\left(i\varepsilon \right)+\frac{\pi_{f}\cdot \varepsilon }{A\cdot B_{f}\left(\tau \right)}\sum_{r=1}^{M}[\left(A_{1}^{r}+A_{2}^{r}+A_{3}^{r}+A_{4}^{r}\right)\\ +\left(A_{5}+A_{6}+A_{7}+A_{8}\right)], \end{array}$$

where

$$\begin{array}{c} A_{1}^{r}=\Delta_{1,1}^{f,r}\left(i\varepsilon \right)\cdot y_{1,f}\left(i\varepsilon \right)\cdot y_{1,r}\left(i\varepsilon \right)\cdot \Omega_{f,r}\cdot \left(p_{1,1,1}^{f,r}-1\right)\leq 0,\\ A_{2}^{r}=\Delta_{1,2}^{f,r}\left(i\varepsilon \right)\cdot y_{1,f}\left(i\varepsilon \right)\cdot y_{2,r}\left(i\varepsilon \right)\cdot \Omega_{f,r}\cdot \left(p_{1,2,1}^{f,r}-1\right)\leq 0,\\ A_{3}^{r}=\Delta_{2,1}^{f,r}\left(i\varepsilon \right)\cdot y_{2,f}\left(i\varepsilon \right)\cdot y_{1,r}\left(i\varepsilon \right)\cdot \Omega_{f,r}\cdot p_{2,1,1}\geq 0,\\ A_{4}^{r}=\Delta_{2,2}^{f,r}\left(i\varepsilon \right)\cdot y_{2,f}\left(i\varepsilon \right)\cdot y_{2,r}\left(i\varepsilon \right)\cdot \Omega_{f,r}\cdot p_{2,2,1}\geq 0,\\ A_{5}=\Delta_{1,1}^{f,M+1}\left(i\varepsilon \right)\cdot y_{1,f}\left(i\varepsilon \right)\cdot u_{1,f}\left(i\varepsilon \right)\cdot \Omega_{f,M+1}\left(i\varepsilon \right)\cdot \left(p_{1,1,1}^{f,M+1}-1\right)\leq 0,\\ A_{6}=\Delta_{1,2}^{f,M+1}\left(i\varepsilon \right)\cdot y_{1,f}\left(i\varepsilon \right)\cdot u_{2,f}\left(i\varepsilon \right)\cdot \Omega_{f,M+1}\left(i\varepsilon \right)\cdot \left(p_{1,2,1}^{f,M+1}-1\right)\leq 0,\\ A_{7}=\Delta_{2,1}^{f,M+1}\left(i\varepsilon \right)\cdot y_{2,f}\left(i\varepsilon \right)\cdot u_{1,f}\left(i\varepsilon \right)\cdot \Omega_{f,M+1}\left(i\varepsilon \right)\cdot p_{2,1,1}^{f,M+1}\geq 0,\\ A_{8}=\Delta_{2,2}^{f,M+1}\left(i\varepsilon \right)\cdot y_{2,f}\left(i\varepsilon \right)\cdot u_{2,f}\left(i\varepsilon \right)\cdot \Omega_{f,M+1}\left(i\varepsilon \right)\cdot p_{2,2,1}^{f,M+1}\geq 0. \end{array}$$

Because we want to maximize the value of $y_{1,f}$ and because we can vary each of the terms $A_{1}^{r}$–$A_{8}^{r}$ independently by tuning an appropriate control parameter (without changing the others), the following values of the control parameters will ensure the maximum of the state variable $y_{1,f}$ at time (i+1)ε:

$$\Delta^{f,r}(i\varepsilon )=\left(\begin{array}{cc} \Delta^{min} & \Delta^{min}\\ \Delta^{max} & \Delta^{max} \end{array}\right),\;\Delta^{f,M+1}(i\varepsilon )=\left(\begin{array}{cc} \Delta^{min} & \Delta^{min}\\ \Delta^{max} & \Delta^{max} \end{array}\right).$$

We also notice that our selection of the control parameters does not affect the values of the state variables $y_{1,r}$ at time (i+1)ε for r≠f. This observation completes the proof of this theorem. □

## 10. Numerical Experiments

To corroborate our findings, we performed numerical tests. In these tests, we solved the control problem at stake using two well-established numerical algorithms: the Forward–Backward Sweep method and the Direct method [76], and compared—whenever it is possible—their outputs against controllers (26) and (31). All code necessary to replicate our experiments is available in the Supplementary Materials.

Starting from an initial guess control, the Forward–Backward Sweep method (henceforth referred to as the FBS method) consecutively repeats the following steps: (i) it solves the master equation, Equation (8), (ii) integrates the Euler–Lagrange Equation (16), thereby finding the adjoint function, and (iii) maximizes the resulting Hamiltonian–Pontryagin function pointwise. During these iterations, the method either converges—not necessarily to an optimal solution—or loops/diverges.

The Direct method simply boils down the control problem at stake to a constrained optimization problem, where all the values of the control function on a grid are subject to variation.

To investigate the effect of the initial guess on the output of these numerical algorithms, we used a multi-start approach, with the following constant uniform control functions as the inputs of the algorithms: $\Delta \left(\tau \right)\equiv \Delta_{min},\Delta \left(\tau \right)\equiv \Delta_{*},$ and $\Delta \left(\tau \right)\equiv \Delta_{max}$, where $\Delta_{min}\leq \Delta_{*}\leq \Delta_{max}$. For our tests, we set $\Delta_{min}=0.5$ and $\Delta_{max}=1$. We applied $\Delta_{*}=0.75$ to get a control function that lies “between” extreme controllers $\Delta_{min}$ and $\Delta_{max}$. We also used this value as a default in situations when singular control components cannot be obtained by differentiating the partial derivatives of the Hamiltonian–Pontryagin function.

We also employed the controllers from (26) and (31) as starting guesses in simulations with a two-element opinion alphabet (m=2) to check whether the FBS method converges on them.

### 10.1. Some Details on Model Parameters Calibration

We orchestrate our experiments in accordance with the size *m* of the opinion alphabet. We consider the cases m=2, m=3, and m=5.

We departed from the case m=2, for which we have the controllers in (26) and (31). For our tests, we employed two collections of transition probability tables. The first one was motivated by the empirically calibrated transition probability table in (5) (see Section 4.7). To be more specific, we preserved the main patterns of (5) and created two new transition probability tables:

(32) $$P_{1}=\left(\begin{array}{cc} 0.99 & 0.01\\ 0.95 & 0.05 \end{array}\right),P_{2}=\left(\begin{array}{cc} 0.07 & 0.93\\ 0.03 & 0.97 \end{array}\right),$$

(33) $$P_{1}=\left(\begin{array}{cc} 0.98 & 0.02\\ 0.96 & 0.04 \end{array}\right),P_{2}=\left(\begin{array}{cc} 0.06 & 0.94\\ 0.04 & 0.96 \end{array}\right)$$

so that the table in (32) gives rise to higher (vs. (33)) levels of conformity (copying the opinion of a partner) and lower levels of anti-conformity (changing the opinion after a conversation with a like-minded individual) [63]. In accordance with previous empirical studies on influence in social groups [57], we employed (32) in in-type interactions and (33) in out-type interactions.

The second collection of transition probability tables was created artificially, with no direct reference to any empirical data, but indicating two well-known tendencies of people—the tendency for conformity [77] and the tendency to incline towards the current opinion [64]:

(34) $$P_{1}=\left(\begin{array}{cc} 1 & 0\\ 0.7 & 0.3 \end{array}\right),P_{2}=\left(\begin{array}{cc} 0.3 & 0.7\\ 0 & 1 \end{array}\right).$$

(35) $$P_{1}=\left(\begin{array}{cc} 1 & 0\\ 0.9 & 0.1 \end{array}\right),P_{2}=\left(\begin{array}{cc} 0.1 & 0.9\\ 0 & 1 \end{array}\right).$$

It is worth noting that, in contrast to (32) and (33), the tables in (34) and (35) are symmetric. The table in (34), which exhibits higher conformity rates, was applied to in-type interactions, and (35) was applied to out-type interactions.

To investigate the control problem in the case of a three-element opinion alphabet (m=3), we considered variant transition probability tables that encode different social influence mechanisms established in the literature, such as assimilative influence or bounded confidence (see [7,8,19] for details). Since our findings on the performances of the numerical methods were virtually the same, we would like to focus on a collection of two transition probability tables that were generated using the large language model *Perplexity*, for which we obtained the effect of varying the initial guess on the output of the Direct method. The resulting tables are provided in Appendix A.3. Note that these tables, as well as those obtained for m=5, regard opinions as ordinal social objects. Therefore, we can speak about the underlying opinion alphabet as a one-dimensional opinion spectrum, with $Z_{1}$ and $Z_{m}$ representing its extremes.

**Figure A1:**
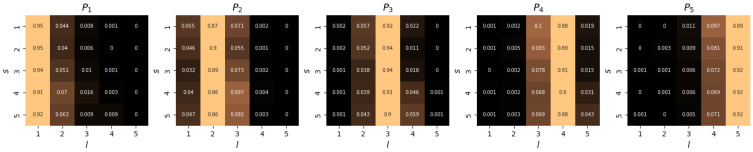
Transition probability table for a 5-element opinion alphabet derived from the empirical data of [21].

While dealing with a five-element opinion alphabet (m=5), we harnessed the transition probability table from [7], which was calibrated using empirical longitudinal data from an online social network (N∼ 1,500,000). This allowed us to optimize the ranking algorithm in real-world settings. Due to the large size of the corresponding transition table, we portray it in a separate figure—see Figure A1 in Appendix B. We refer the interested Reader to Refs. [7,21] for a detailed analysis of the social influence patterns encoded by this table and the underlying social data. We also made use of the empirical data from [78]. This longitudinal dataset describes the opinion dynamics of a large sample of users N∼ 30,000 and includes information about individual characteristics other than opinion, such as gender. This allowed us to investigate the role of gender in recommendations.

In the results presented below, one cell of the grid corresponds to one Monte Carlo step (one unit of time τ), which, in turn, refers to *N* steps in the initial discrete time *t*.

We are now in a position to present the results of our numerical experiments. We start from the case m=2.

### 10.2. Results: M=2

**Figure 3:**
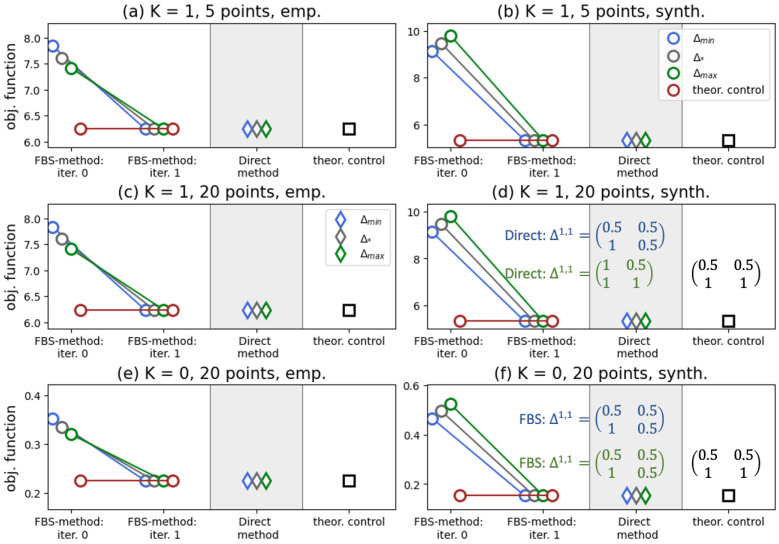
We investigate the performances of the FBS method and the Direct method against our analytically derived controller, (26). The opinion weight vector is v=(0,1) (we minimize the presence of opinion $Z_{2}$ in the system). We test two collections of transition probability tables: (i) (32) and (33) (left panels) and (ii) (34) and (35) (right panels). We also vary the size of the grid and the value of *K*. In this figure and in the ongoing ones, various colors signify different initial guesses, as marked in the legend. In panels (**d**,**f**), we plot controllers derived by the numerical algorithms for initial guesses $\Delta_{min}$ and $\Delta_{max}$ (the colors are aligned with the legend). Simulation issues are detailed in Appendix A.1.

Figure 3 indicates that our solution in (26) yields the best performance in the case M=1, as do the FBS and Direct methods. We report that, for synthetic transition probability tables (panels (b), (d), and (f)), a singular control appears in the iterations of the FBS method. In the experiments presented in these three panels, we found that the outcomes of the numerical methods depend on the initial guess and deviate from the controller in (26), as shown in panels (d) and (f). We should say here that the Direct method is quite sensitive to the grid size, and on a grid of 20 points, depending on the initial guess, it may require ∼4 min to converge (all experiments were performed on Dell PowerEdge R740, 2× Intel Xeon Gold 5218R, 320 Gb RAM), whereas the FBS method consistently converges in two iterations, and it usually takes ∼0.01 s.

**Figure 4:**
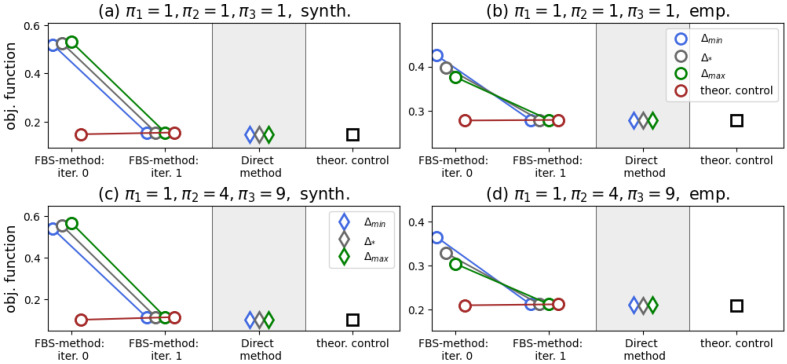
We consider a system with m=2 opinions and M=3 types of authentic agents, with in-type interactions being described by the table in (34) (left panels) or (32) (right panels), and out-type communications being defined by the table in (35) (left panels) or (33) (right panels). All simulations were performed on a grid of 5 points, with K=0. In these simulations, we also varied the values of the activity parameters, as shown in the panel titles. Simulation issues are detailed in Appendix A.2.

The aforementioned findings generally remain valid when considering the case of more than one type of native agent. In Figure 4, we show the results of numerical tests for M=3 of such types. From this figure, one can observe the absence of any effect of varying the activity parameters on the performance of the algorithms. Again, we see that the FBS method, the Direct method, and our controller yield the same value of the objective functional, with the FBS method requiring two iterations for convergence.

### 10.3. Results: M=3

For the case of a three-element opinion alphabet, our analytically derived controllers are meaningless and cannot be applied. However, we can still make use of the FBS method and the Direct method. As well, we can try to find any similarities between the outputs of the numerical algorithms and the controllers from (26) and (31) (see Remark 3).

Our current foci are two core scenarios (m=3,M=2). In the first one, the system starts from the state of

$$q=\left(\begin{array}{cc} 0.4 & 0\\ 0.1 & 0.1\\ 0 & 0.4 \end{array}\right),$$

which indicates that opinions and types are strongly correlated: agents with type $\Xi_{1}$ tend to have opinion $Z_{1}$, and agents with type $\Xi_{2}$ are inclined to opinion $Z_{3}$. To the contrary, in the second scenario, the starting state is given by

$$q=\left(\begin{array}{cc} 0.2 & 0.2\\ 0.1 & 0.1\\ 0.2 & 0.2 \end{array}\right),$$

which shows no correlation between opinions and types. Both of these scenarios concern an initially polarized population, and we set the opinion weight vector as v=(1,0,1)—thus said, the goal of stewardship is to depolarize the community.

**Figure 5:**
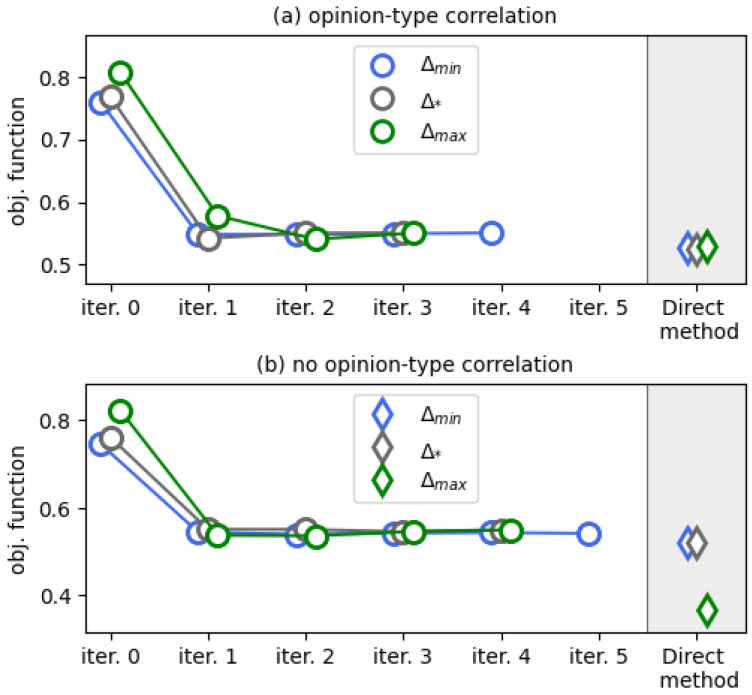
Results of numerical experiments for a system with opinions and types being correlated (**a**) and for an uncorrelated system (**b**). The ticks “iter. *i*” show the working of the FBS method. Simulation issues are detailed in Appendix A.3.

We present our results in Figure 5. In contrast to the case m=2, we now see that the Direct method slightly outperforms the FBS method, which now takes much more than two iterations to converge. All the iterations of the FBS method depicted in Figure 5 were accompanied by the presence of singular control components.

**Figure 6:**
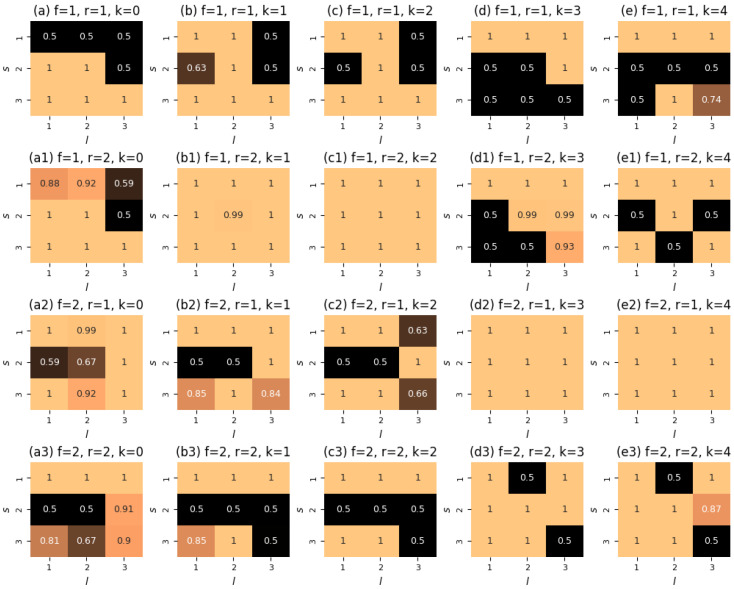
Organization of the controller obtained by the Direct method with the starting guess $\Delta \left(\tau \right)\equiv \Delta_{max}$ in the no-correlation scenario (see Figure 5b). Each panel shows a slice $\Delta^{f,r}$ (the indices are provided in the titles) of the function Δ(τ) defined on a 5-point grid (marked by *k*).

The interesting point of Figure 5b is that we acknowledge the effect of the initial guess on the performance of the Direct method: when starting from $\Delta (\tau )\equiv \Delta_{max}$, the numerical method yields the lowest value of the objective functional. In Figure 6, we show the corresponding control function. If this controller were to follow the principles of the controllers of Theorems 1 and 2 (see Remark 3), then one would expect that each slice $\Delta^{f,r}$ has the following organization: the components in the second row of $\Delta^{f,r}$ are equal to $\Delta_{min}$ (because we would like to maximize the presence of opinion $Z_{2}$), whereas the components of the first and third rows are equal to $\Delta_{max}$ (as we would like to suppress radical opinions $Z_{1}$ and $Z_{3}$ from the system). However, this seems to be the case only for a few of the panels, and even then only partially.

However, the controller derived by the Direct method with $\Delta (\tau )\equiv \Delta_{min}$ as a starting guess is much closer to this intuition—see Figure A3 (Appendix B). At least, most of its slices have zero-valued second rows. Nonetheless, one can notice a reasonable level of asymmetry in this control function, which is manifested in opinion $Z_{3}$ being closed for contacts. The output derived from the FBS method displays an opposite asymmetry pattern, where opinion $Z_{1}$ is underrepresented in interactions (see Figure A4 in Appendix B). Comparing this controller with the one obtained by the FBS method in the no-correlation setting (plotted in Figure A5, Appendix B), we see that the presence of opinion-type correlations sufficiently affects the organization of the controller and makes it more inclined to prohibit any contacts with opinion $Z_{3}$.

### 10.4. Results: M=5

We finalize our analysis with the case m=5. Because the number of control parameters to estimate at each grid point grows quadratically with both *m* and *M*, we focused on scenarios with M=1 and M=2 types of native agent.

For M=1, we investigated two stylized scenarios: (i) v=(2,1,0,1,2) (depolarization of the system) and (ii) v=(4,3,2,1,0) (agents’ opinions are steered towards the right endpoint of the opinion spectrum—opinion nudging [2]). In the case of the first scenario, the initial system state was

$$q={\left(\begin{array}{ccccc} 0.3 & 0.15 & 0.1 & 0.15 & 0.3 \end{array}\right)}^{T},$$

(a perfectly symmetric polarized social system).

For the second scenario, the starting point was

$$q={\left(\begin{array}{ccccc} 0.7 & 0.2 & 0.1 & 0 & 0 \end{array}\right)}^{T},$$

which represents a social system inclined to the left side of the opinion spectrum.

**Figure 7:**
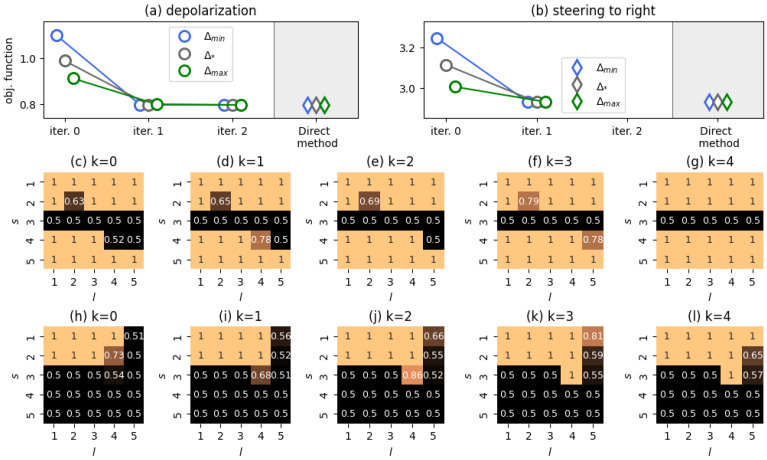
Plotted are the results of our numerical experiments for m=5,M=1. The ticks “iter. *i*” in panels (**a**,**b**) show the workings of the FBS method. Panels (**c**–**g**) show the controller derived by the Direct method (starting guess $\Delta \left(\tau \right)\equiv \Delta_{min}$) for the depolarization scenario (**a**). Panels (**h**–**l**) show the controller derived by the Direct method (starting guess $\Delta \left(\tau \right)\equiv \Delta_{min}$) for the opinion nudging scenario (**b**). Simulation issues are detailed in Appendix A.4.

Our results are summarized in Figure 7. We see that the Direct method yields the same quality as the FBS method in both scenarios, with no effects of the initial guess on the outputs of the algorithms. The resulting outputs of the Direct method are also presented in the figure. One can notice that they largely follow the intuition of Theorems 1 and 2: an opinion to be minimized should communicate more often, whereas a desirable opinion should avoid any interactions.

**Figure 8:**
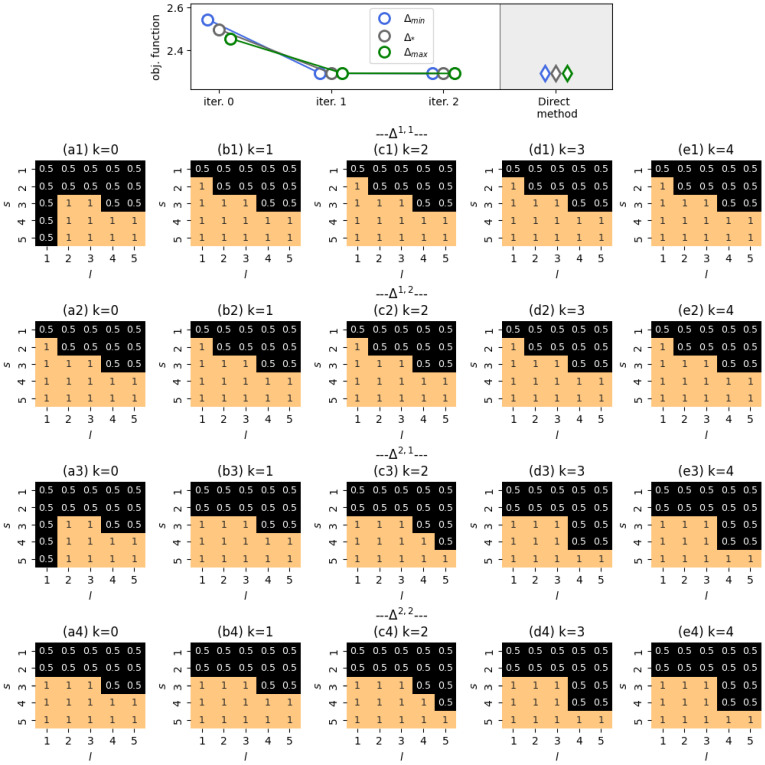
Plotted are the results of our numerical experiments for m=5,M=2. The upper panel shows the convergence of the FBS method across various initial guesses, indicating that the resulting controllers are as optimal as those obtained using the Direct method, though they differ slightly. The subsequent panels demonstrate the control achieved by the FBS method. Simulation issues are detailed in Appendix A.5.

**Figure A2:**
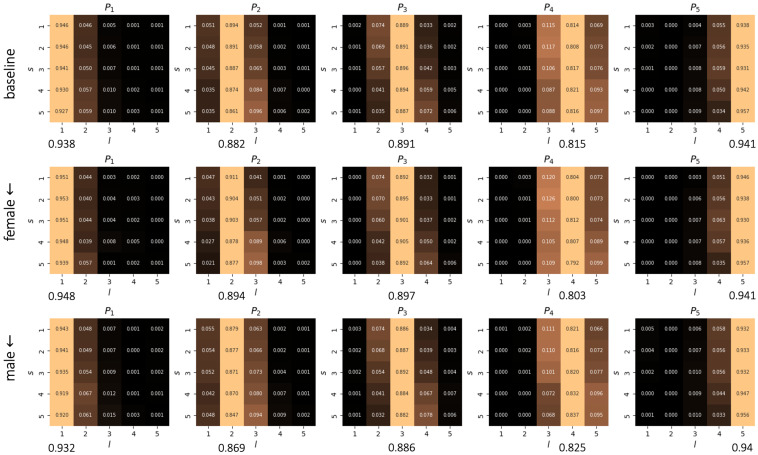
Transition probability tables for a 5-element opinion alphabet derived from the empirical data from [78]. The upper table was calibrated using the entire dataset and is not informed about individual characteristics. The middle table was calibrated using a subset of the data where female individuals were influenced. The lower table was created for the subsample where male individuals were influence objects. For each table, we provide the rates of resistance to influence as averages of $p_{s,l,s}$ over l∈[m] for fixed *s* (each value is depicted under the corresponding column). From these rates, one of the main findings of Ref. [78] can be seen: women with conservative opinions are more resistant to influence than men with conservative views.

**Figure A3:**
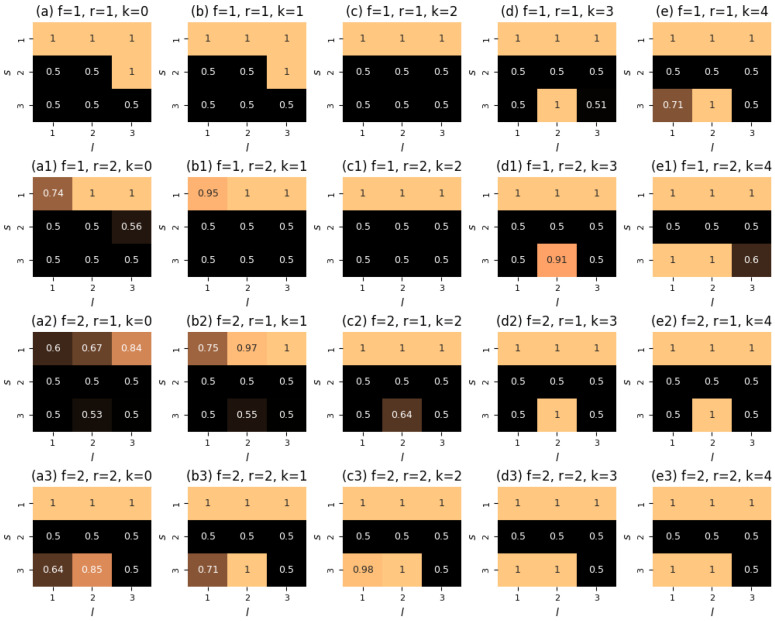
We plot the open-loop controller obtained by the Direct method with the starting guess $\Delta \left(\tau \right)\equiv \Delta_{min}$ in the no-correlation scenario (see Figure 5b).

**Figure A4:**
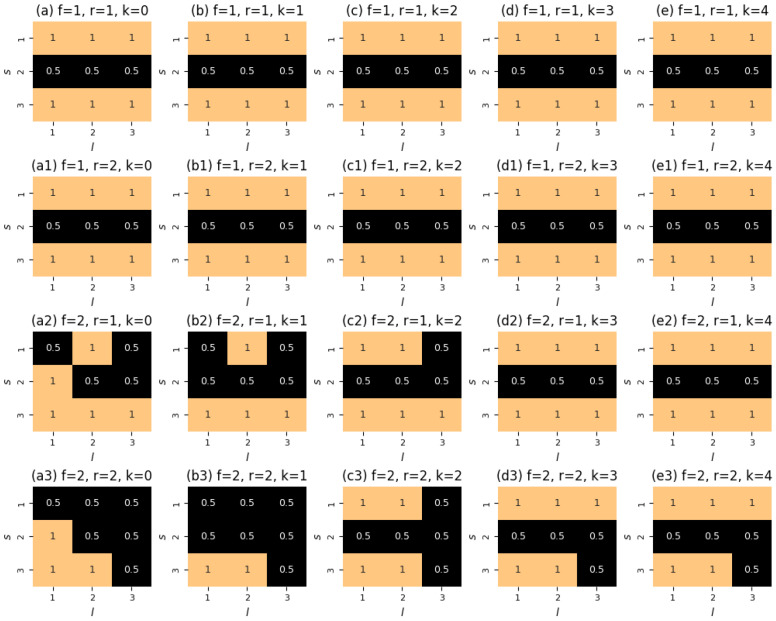
We plot the open-loop controller obtained by the FBS method with the starting guess $\Delta \left(\tau \right)\equiv \Delta_{min}$ in the no-correlation scenario (see Figure 5b).

**Figure A5:**
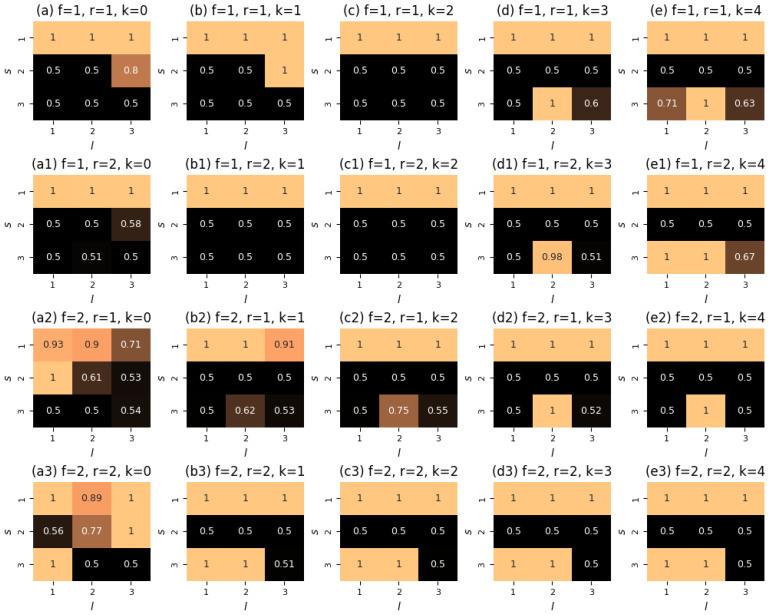
We plot the open-loop controller obtained by the FBS method with the starting guess $\Delta \left(\tau \right)\equiv \Delta_{min}$ in the opinion-type correlation scenario (see Figure 5a).

The case M=2 was explored under the setting that agents’ opinions should shift to the left, as indicated by v=(0,1,2,3,4). The initial state of the system was

$$q={\left(\begin{array}{ccccc} 0 & 0.05 & 0.1 & 0.1 & 0.25\\ 0.05 & 0.1 & 0.15 & 0.1 & 0.1 \end{array}\right)}^{T},$$

which shows a general bias towards the right end of the opinion spectrum, with females ($\Xi_{1}$) being more inclined than males ($\Xi_{2}$). The results of our experiments are shown in Figure 8. We see that the FBS-derived controller hinders communications in which the influence source has an opinion that is more to the right of the influence target, thus preventing the target’s opinion from moving towards the right. We also notice that this pattern is more pronounced for interactions in which male agents are influenced. This can be explained by the observation that female agents with left-leaning opinions tend to be less susceptible to influence than male agents with the same opinions, as shown in Figure A2 (Appendix B). As such, we conclude that the ranking algorithm—which strives for the left side of the opinion spectrum—should shield such male agents from the influence of right-leaning opinions.

From panels (a1) and (a3) of Figure 8, one can notice that if a female agent is the source of influence, then, in the beginning of the system’s evolution, agents with opinion $Z_{1}$ should not influence other agents. This is simply due to the absence of any female agents with opinion $Z_{1}$ at τ=0, as specified by the initial state *q*.

## 11. Conclusions

This paper proposes a model-dependent theoretical framework for finding an optimal design of a ranking algorithm to affect individuals’ opinions. Building upon the mean-field approximation of a nonlinear opinion dynamics model [58], we formulated a control problem in which the time-dependent parameters of a ranking algorithm are dynamically adjusted to achieve a desirable opinion distribution.

We proved that the control problem at stake has a solution. Using the Pontryagin Maximum Principle, we characterized certain properties of optimal controllers. After applying finite-difference schemes to the control problem, we solved it for the case of a two-element opinion alphabet and an arbitrary number of agent types. The resulting solution is intuitive: if there are only two possible opinions, then one should keep the agents holding a “desirable” opinion away from any contacts to prevent them from having any possibility of changing their opinion. And, on the contrary, one should facilitate communications between individuals with an “undesirable” opinion—to maximize the likelihood of opinion updating among these agents. What is important here is that this control does not depend on external factors, such as social bots and their attacks.

We conducted extensive numerical tests to bolster our theoretical findings. We found that the finite-difference scheme controllers yield the same quality as those derived from several established numerical methods. Our experiments also spanned the cases of three- and five-element opinion alphabets. We examined two stylized scenarios: the depolarization of an initially polarized society and the nudging of a social system towards a given edge of an opinion spectrum. Social systems with node-level and edge-level correlations were covered in our simulations, and we acknowledge the effect of such correlations on the outputs of the numerical algorithms. The obtained controllers tended to be of a boundary-bang type [71], which is due to the fact that our model is linear with respect to the control variables.

We recognize that our approach is not without limitations. Our operationalization of the ranking algorithm involves agents avoiding interactions with certain probabilities. However, in a real setting, a user who is deprived of a piece of content by an algorithm will replace it with a different piece of content. Next, we assume that the parameters of the ranking algorithm are adjusted independently: the lower and upper bounds are the only constraints we impose on them. However, it is likely not the case for real-world online platforms. Effectively, it would be more realistic to assume that there is a total constraint on the number of interactions blocked by the system, with the constraint stemming from hardware capabilities. Therefore, the set of admissible controls should be a simplex, rather than a hyperrectangle. It is also worth noting that our model omits one of the key aspects of platform behavior: the pursuit of increased user engagement alongside opinion nudging [5].

Nonetheless, the theoretical framework proposed is flexible enough to embrace information on agents’ attributes, their activity heterogeneity, and the mesoscopic and macroscopic properties of the underlying networks, including modularity patterns. Our control model covers different mechanisms of social influence, thus providing an opportunity to exert control over ranking algorithms in the presence of inevitable uncertainty regarding the true nature of social influence [25].

## Data Availability

The original contributions presented in this study are included in the article. Further inquiries can be directed to the corresponding author.

## Supplementary Materials

The following supporting information can be downloaded at: https://www.mdpi.com/article/10.3390/e28030333/s1.

## Appendix A. Simulation Details

### Appendix A.1. Simulation Details for Figure 3

The case m=2,M=1 is considered. The starting point of the dynamical system is given by $q=\left(\begin{array}{c} 0.5\\ 0.4 \end{array}\right)$, the bots’ behavior (whose population is 0.1) is described by $u_{2,1}\left(\tau \right)\equiv 0.1$. That is, bots consistently disseminate opinion $Z_{2}$ all the time. The network configuration is given by $\rho_{1,1}=1,\rho_{1,2}=0.5$ (ties between authentic agents appear twice as often as those between authentic agents and bots). The activity parameters are $\pi_{1}=2,\pi_{2}=3$, which means that bots are more active than native agents. The opinion weight vector is V=(0,1): the presentation of opinion $Z_{2}$—which is disseminated by bots—should be minimized. Interactions between native agents are described either by table (32) (see the left panels of the figure), or by table (34) (right panels). Interactions between native agents and bots are portrayed by transition probability tables (33) (left panels) and (35) (right panels).

### Appendix A.2. Simulation Details for Figure 4

In this figure, the scenario m=2,M=3 is presented. The starting point of the dynamical system is

$$q=\left(\begin{array}{ccc} 0.3 & 0.2 & 0\\ 0 & 0.2 & 0.3 \end{array}\right),$$

which means that there are no bots in the system. The network configuration is given by

(A1) $$\begin{array}{c} \rho_{1,1}=0.3,\rho_{1,2}=0.1,\rho_{1,3}=0.1,\\ \rho_{2,1}=0.1,\rho_{2,2}=0.4,\rho_{2,3}=0.1,\\ \rho_{3,1}=0.1,\rho_{3,2}=0.1,\rho_{3,3}=0.5. \end{array}$$

The parameters of the stochastic block model reflect the phenomenon of homophily in social networks—the tendency of individuals to contact those who are similar [79]. In our case, we refer to similarity in terms of agent types—agents with the same type have more chances to be connected by an edge. The parameters in (A1) also indicate that such a tendency is more pronounced among the agents of type $\Xi_{2}$ and, especially, $\Xi_{3}$. The activity parameters are varied in experiments, as shown in the captions of the panels of Figure 4. The opinion weight vector is V=(0,1). The transition probability tables employed in the referenced experiments are the same as those listed in the previous subsection of the appendix.

### Appendix A.3. Simulation Details for Figure 5

Here, we present a transition probability table calibrated by the large language model *Perplexity* for m=3,M=2. As a focal question, we considered attitudes towards anime. Three opinion values were introduced (a three-point Likert scale):

- $Z_{1}$—positive attitude;
- $Z_{2}$—neutral attitude;
- $Z_{3}$—negative attitude.

Using prompt-engineering, we asked *Perplexity* to evaluate its opinion on the focal question, given that the following information is provided:

- The model’s previous opinion (measured on the same scale);
- The opinion of its “friend” (measured on the same scale);
- The model’s “gender”—we asked the model to roleplay either a male person ($\Xi_{1}$) or a female person ($\Xi_{2}$).

With this information being digested by *Perplexity* via in-context learning, the model outputs its “new” opinion, which can then be employed in the calibration of transition probability tables.

We obtained the following tables:

$$P_{1}^{1,1}=\left(\begin{array}{ccc} 0.94 & 0.05 & 0.01\\ 0.7 & 0.25 & 0.05\\ 0.65 & 0.25 & 0.1 \end{array}\right),P_{2}^{1,1}=\left(\begin{array}{ccc} 0.45 & 0.5 & 0.05\\ 0.25 & 0.65 & 0.1\\ 0.15 & 0.25 & 0.6 \end{array}\right),P_{3}^{1,1}=\left(\begin{array}{ccc} 0.35 & 0.45 & 0.2\\ 0.15 & 0.45 & 0.4\\ 0.05 & 0.15 & 0.8 \end{array}\right),$$

$$P_{1}^{1,2}=\left(\begin{array}{ccc} 0.92 & 0.07 & 0.01\\ 0.8 & 0.15 & 0.05\\ 0.7 & 0.2 & 0.1 \end{array}\right),P_{2}^{1,2}=\left(\begin{array}{ccc} 0.35 & 0.55 & 0.1\\ 0.2 & 0.7 & 0.1\\ 0.2 & 0.35 & 0.45 \end{array}\right),P_{3}^{1,2}=\left(\begin{array}{ccc} 0.25 & 0.5 & 0.25\\ 0.1 & 0.35 & 0.55\\ 0.08 & 0.22 & 0.7 \end{array}\right),$$

$$P_{1}^{2,1}=\left(\begin{array}{ccc} 0.89 & 0.1 & 0.01\\ 0.7 & 0.2 & 0.05\\ 0.45 & 0.3 & 0.25 \end{array}\right),P_{2}^{2,1}=\left(\begin{array}{ccc} 0.5 & 0.45 & 0.05\\ 0.22 & 0.68 & 0.1\\ 0.1 & 0.15 & 0.75 \end{array}\right),P_{3}^{2,1}=\left(\begin{array}{ccc} 0.4 & 0.25 & 0.35\\ 0.25 & 0.5 & 0.25\\ 0.1 & 0.25 & 0.65 \end{array}\right),$$

$$P_{1}^{2,2}=\left(\begin{array}{ccc} 0.87 & 0.12 & 0.01\\ 0.65 & 0.3 & 0.05\\ 0.55 & 0.25 & 0.2 \end{array}\right),P_{2}^{2,2}=\left(\begin{array}{ccc} 0.55 & 0.4 & 0.05\\ 0.28 & 0.6 & 0.12\\ 0.12 & 0.18 & 0.7 \end{array}\right),P_{3}^{2,2}=\left(\begin{array}{ccc} 0.2 & 0.55 & 0.25\\ 0.15 & 0.5 & 0.35\\ 0.07 & 0.18 & 0.75 \end{array}\right).$$

From these tables, one can notice a sufficient level of asymmetry, a presence of dissimilative opinion shifts (see matrices $P_{2}^{f,r}$), and the absence of bounded confidence patterns—for a fixed non-central opinion ($Z_{1}$ or $Z_{3}$), the probability of the opinion being unchanged exhibits a monotonic decay with opinion distance and shows no tendency to increase in the case of communications with the opposite opinion (see matrices $P_{1}^{f,r}$ and $P_{3}^{f,r}$—the components $p_{1,3,1}^{f,r}$ and $p_{3,1,3}^{f,r}$, respectively). Generally, these tables display a strong tendency towards assimilation, in line with research on the behavior of large language models [80].

### Appendix A.4. Simulation Details for Figure 7

The case m=5,M=1 is considered. The starting point of the dynamical system is either

$$q={\left(\begin{array}{ccccc} 0.3 & 0.15 & 0.1 & 0.15 & 0.3 \end{array}\right)}^{T},$$

(panel (a)), or

$$q={\left(\begin{array}{ccccc} 0.7 & 0.2 & 0.1 & 0 & 0 \end{array}\right)}^{T},$$

(panel (b)). In both cases, there are no bots in the system

In panel (a), we show a depolarization scenario, which is given by the weight-vector v=(2,1,0,1,2). In panel (b), the purpose of stewardship is to steer the system towards the right end-point of the opinion scale (v=(4,3,2,1,0)). We set K=0 and consider a five-point grid. Figure A1 plots the underlying transition probability table (see the next subsection of the appendix). This table was calibrated on the empirical data from [21], as described in Ref. [7].

### Appendix A.5. Simulation Details for Figure 8

The case m=5,M=2 was considered. The starting point of the dynamical system was

$$q={\left(\begin{array}{ccccc} 0 & 0.05 & 0.1 & 0.1 & 0.25\\ 0.05 & 0.1 & 0.15 & 0.1 & 0.1 \end{array}\right)}^{T}.$$

The network configuration is given by

$$\begin{array}{c} \rho_{1,1}=0.4,\;\rho_{1,2}=0.1,\\ \rho_{2,1}=0.1,\;\rho_{2,2}=0.4. \end{array}$$

We used transition tables obtained by virtue of the empirical longitudinal data from Ref. [78], see Figure A2 (the medium and bottom panels). These tables show how female ($\Xi_{1}$) and male ($\Xi_{2}$) users respond to influence, regardless of the gender of the influence source (this covariate was found to be statistically insignificant in [78]). We used the middle table to calibrate $P^{1,1}$ and $P^{1,2}$ and the bottom table to calibrate $P^{2,1}$ and $P^{2,2}$, respectively.

## Appendix B. Supporting Figures

In this section, we present the auxiliary figures, Figure A1–Figure A5.
